# Atractylenolide-I Sensitizes Triple-Negative Breast Cancer Cells to Paclitaxel by Blocking CTGF Expression and Fibroblast Activation

**DOI:** 10.3389/fonc.2021.738534

**Published:** 2021-10-06

**Authors:** Meng Wang, Xue-Zhen Li, Ming-Xing Zhang, Qian-Yu Ye, Ying-Xia Chen, Xu Chang

**Affiliations:** ^1^ First Department of Surgery, Panyu Hospital of Chinese Medicine, Guangzhou, China; ^2^ Department of Breast Surgery, Guangdong Second Hospital of Traditional Chinese Medicine, Affiliated Hospital of Guangzhou University of Chinese Medicine, Guangzhou, China; ^3^ Department of Mammary Disease, Panyu Hospital of Chinese Medicine, Guangzhou, China

**Keywords:** CTGF, fibroblast, chemoresistance, microenvironment, paclitaxel, triple-negative breast cancer, atractylenolide-I

## Abstract

This investigation was conducted to elucidate whether atractylenolide-I (ATL-1), which is the main component of Atractylodes macrocephala Koidz, can sensitize triple-negative breast cancer (TNBC) cells to paclitaxel and investigate the possible mechanism involved. We discovered that ATL-1 could inhibit tumor cell migration and increase the sensitivity of tumor cells to paclitaxel. ATL-1 downregulated the expression and secretion of CTGF in TNBC cells. Apart from inhibiting TNBC cell migration *via* CTGF, ATL-1 downregulated the expression of CTGF in fibroblasts and decreased the ability of breast cancer cells to transform fibroblasts into cancer-associated fibroblasts (CAFs), which in turn increased the sensitivity of TNBC cells to paclitaxel. In a mouse model, we found that ATL-1 treatments could enhance the chemotherapeutic effect of paclitaxel on tumors and reduce tumor metastasis to the lungs and liver. Primary cultured fibroblasts derived from inoculated tumors in mice treated with ATL-1 combined with paclitaxel expressed relatively low levels of CAF markers. Collectively, our data indicate that ATL-1 can sensitize TNBC cells to paclitaxel by blocking CTGF expression and fibroblast activation and could be helpful in future research to determine the value of ATL-1 in the clinical setting.

## Introduction

Breast cancer is one of the most common malignant tumors among women worldwide, and it is increasingly being identified in relatively young populations, while its mortality rate is increasing annually (1). Although hormone therapy, chemotherapy, and targeted therapy have significantly improved efficacy in breast cancer patients, chemotherapy resistance is still a substantial challenge in the face of triple-negative breast cancer (TNBC), for which hormone therapy is not available. Chemotherapy resistance is becoming increasingly common and involves a variety of mechanisms, including P-glycoprotein-dependent drug efflux, DNA damage and repair, epigenetic changes and apoptosis disorders (2). In these mechanisms, cancer-associated fibroblasts (CAFs), which are activated fibroblasts, also play an important role in promoting the drug resistance of tumor cells in the tumor microenvironment (3). CAFs can lead to chemotherapy resistance in tumor cells by secreting exosomes and cytokines, such as IL-6, IL-8 and HGF (4–8). CAFs can create obstacles to chemotherapeutic drug delivery. CAFs can lead to leakage in tumor blood vessels and decrease the concentration of intravenous drugs (9). In addition, CAFs can also increase collagen secretion in solid tumors such as pancreatic ductal adenocarcinoma (PDAC) and breast cancer, enhance the density of tumor tissues, reduce vascular density, and pressure the tumor vascular system (10, 11).

Atractylenolide-I (ATL-1) is a naturally occurring sesquiterpene lactone isolated from Atractylodes macrocephala Koidz [Family: Compositae] (12, 13) that has been applied in anti-inflammatory, antifibrotic and antitumor treatments (14–18). ATL-1 has antitumor effects on a variety of cancers, including colon cancer (18, 19), breast cancer (17), melanoma (20), ovarian cancer (21, 22) and gastric cancer (23). ATL-1 can inhibit the proliferation of tumor cells (17, 19) and induce apoptosis in tumor cells (18). In addition, ATL-1 was found to reduce the dryness of tumor cells and increase the sensitivity of tumor cells to paclitaxel (22). However, the antitumor mechanism of ATL-1, especially the specific mechanism by which it increases the sensitivity of tumor cells to chemotherapeutic drugs, is still unclear and requires further study. ATL-1 was reported to inhibit fibroblast-myofibroblast differentiation (FMD) and repress fibrosis development in unilateral ureteral obstruction kidneys (16). So we wondered whether ATL-1 could inhibit fibroblast activation in tumor microenvironment.

Connective tissue growth factor (CTGF), also known as CCN2, is a member of the CCN family. Initially, CTGF was found to promote tissue fibrosis and harden tissue structures (24, 25). As a secreted protein, CTGF also plays important roles in tumor cells and the tumor microenvironment. On the one hand, CTGF can promote the proliferation, migration, invasion, EMT and metastasis of tumor cells (26–29); on the other hand, CTGF can induce chemotherapy resistance (30). MDA-MB-231 breast cancer cells with lower CTGF expression are more sensitive to doxorubicin and paclitaxel (31). CTGF can regulate the expression of antiapoptotic genes in tumor cells (32). CTGF can also induce a variety of cells to transform into CAFs in the tumor microenvironment (33–36). CTGF expression is increased in CAFs and negatively correlated with disease-free survival (35). It has also been reported that CTGF can induce autophagy, glycolysis and senescence in MDA-MB-231 breast cancer cells by changing the metabolism of CAFs (37). CAFs have a role in inducing chemotherapy resistance, which explains the mechanism of chemotherapy resistance induction by CTGF from the perspective of the tumor microenvironment.

Our previous clinical trials have proven that Huatan Sanjie decoction based on Atractylodes macrocephala Koidz can significantly increase the sensitivity of patients with TNBC to paclitaxel chemotherapy. Therefore, we examined the effect of ATL-I, which is the main component of Atractylodes macrocephala Koidz, on oncogene expression in MDA-MB-231 TNBC cells by qRT-PCR. We found that ATL-I could significantly reduce the CTGF mRNA level in TNBC cells and fibroblasts. Through functional experiments, we found that ATL- I could significantly reduce the migration of TNBC cells, downregulate the transformation of fibroblasts into CAFs and increase the sensitivity of TNBC cells to paclitaxel. Therefore, we speculated that ATL- I could play a chemotherapy-sensitizing role by downregulating CTGF.

## Materials and Methods

### Chemicals and Antibodies

Atractylenolide-I (AT-I, purity > 98%) and paclitaxel (purity > 98%) were purchased from Selleck Chemicals (Houston, TX, USA). A human CTGF/CCN2 DuoSet ELISA kit and recombinant human CTGF protein (rCTGF) were purchased from R&D Systems (Minneapolis, MN, USA). AT-1 was used at a dose of 0-100 μM (17). rCTGF was used at a dose of 0-20 μg/mL (38). TRIzol reagent was bought from Invitrogen (Carlsbad, CA, USA). M-MLV reverse transcriptase was bought from Promega (Madison, WI, USA). SYBR Green Real-time PCR Master Mix was bought from TOYOBO (Osaka, Osaka Prefecture, Japan). Puromycin was bought from Coolaber Technologies (Beijing, China). Anti-CTGF, anti-αSMA and anti-FAP antibodies were obtained from Abcam (Cambridge, UK). Anti-β-tubulin antibody was obtained from Santa Cruz Biotechnology (Santa Cruz, CA, USA). HRP-conjugated goat anti-rabbit IgG and goat anti-mouse IgG antibodies were obtained from OriGene Technologies (Rockville, MD, USA). Immobilon Western Chemiluminescent HRP Substrate was bought from Millipore (Billerica, MA, USA).

### Cell Lines

MDA-MB-231 cells were purchased from ATCC (Manassas, VA, USA). HS578T cells were purchased from the Cell Resource Center, Institute of Basic Medical Sciences, Chinese Academy of Medical Sciences (Beijing, China). HFF1 and WI-38 cells were purchased from Shanghai Zhong Qiao Xin Zhou Biotechnology (Shanghai, China). MDA-MB-231, HS578T and WI-38 cells were cultured in DMEM supplemented with 10% fetal bovine serum (FBS) (39, 40). HFF1 cells were cultured in DMEM supplemented with 15% FBS. FBS was bought from Gibco (Carlsbad, CA, USA).

### Reverse Transcription and qRT-PCR

Total RNA was isolated using TRIzol reagent according to the manufacturer’s protocol (41). Two microgram of total RNA was reverse transcribed using M-MLV reverse transcriptase. qRT-PCR was performed on ABI StepOne Real-Time PCR System using SYBR Green Real-time PCR Master Mix. The gene-specific primer sequences are listed in **Table 1**. Gene expression levels were normalized to GAPDH. The 2^–ΔΔCt^ method was used for relative quantification (42).

**Table 1:** Primer list for realtime-PCR.

Gene (*Species*)	Forward primer	Reverse primer
CTGF (*Homo sapiens*)	5’-CTTGCGAAGCTGACCTGGAAGA-3’	5’-CCGTCGGTACATACTCCACAGA-3’
FAP (*Homo sapiens*)	5’-GCAGCGACTATGCACAACGA-3’	5’-CCAGAGTGGTGACGGAGACA-3’
α-SMA (*Homo sapiens*)	5’-AGGGCTGTTTTCCCATCCATT-3’	5’-TTTTGCTCTGTGCTTCGTCAC-3’
FAP (*Mus musculus*)	5’-AAAGACCAGGAGATCCACCTT-3’	5’-TGGAGACCACCAAAGAGCATA-3’
α-SMA (*Mus musculus*)	5’-GCCGAGATCTCACCGACTAC-3’	5’-TGTCACGGACAATCTCACGC-3’
GAPDH (*Homo sapiens*)	5’-GTCTCCTCTGACTTCAACAGCG-3’	5’-ACCACCCTGTTGCTGTAGCCAA-3’
GAPDH (*Mus musculus*)	5’-CATCACTGCCACCCAGAAGACTG-3’	5’-ATGCCAGTGAGCTTCCCGTTCAG-3’

### Heatmap for qRT-PCR

To show changes in mRNA levels *via* heatmap, the normalized log2 ratios (mRNA levels with ATL-1 treatment/mRNA levels without ATL-1 treatment) of each gene were calculated. The relative mRNA levels obtained by qRT-PCR were used to calculate the normalized log2 ratios. The calculation process was as follows:

The relative mRNA levels of each gene were calculated by 2^–ΔCt^ method, where


ΔCt=(Ct gene of interest−Ct GAPDH)

Then the log2 ratios (mRNA levels with ATL-1 treatment/mRNA levels without ATL-1 treatment) were as follows (42):


$$\log2\;\text{ratio}=\log2\;(2^{–\Delta \text{Ct with ATL}-1}/2^{–\Delta \text{Ct without ATL}-1})=–\Delta \Delta \text{Ct},$$

where


ΔΔCt=(ΔCt interest gene with ATL−1 treatment−ΔCt the same gene without ATL−1treatment)

It should be noticed that, each of three biological replicates were performed for the group with ATL-1 treatment and the group without ATL-1 treatment. One of the biological replicate in group without ATL-1 treatment was chose as the standard, then the mRNA level of the other two biological replicates without ATL-1 treatment and the three biological replicates with ATL-1 treatment were all calculated the log2 ratio to this standard. The log2 ratio of the standard to itself was zero.

At last, all of the calculated log2 ratios were dealt with Z-score normalization:


$$\text{Z}-\text{score}=({\text{G}}_{\text{i}}–\;\text{mean})/\text{SD}$$

G_i_ means the log2 ratio of one of the replicate of gene G. Mean and SD were calculated using the six calculated log2 ratios of gene G (three in group with ATL-1 treatment and three in group without ATL-1 treatment). The normalized log2 ratio was then shown in different colors in the heatmap, which was drawn by Microsoft Excel 2010 (Redmond, WA, USA). Student’s t test was used to determine the difference between the two groups. The gene-specific primer sequences used in the heatmap are listed in **Supplementary Table S2**.

### Western Blotting

Equal amounts of total protein (20 μg/lane) were separated by SDS-PAGE and transferred to PVDF membranes with a pore size of 0.45 µm (Millipore, Billerica, MA, USA). After blocking with 5% nonfat milk at room temperature for 1 hour, the membranes were incubated at 4°C overnight with primary antibodies against CTGF (1:1000, ab209780), α-SMA (1:5000, ab124964), FAP (1:1000, ab207178) and β-tubulin (1:1000, TA-10), and then with HRP-conjugated goat anti-rabbit IgG (1:2000, ZB-2301) or goat anti-mouse IgG (1:2000, ZB-2305) antibodies for 1 h at room temperature. Finally, Immobilon Western Chemiluminescent HRP Substrate was used to visualize the blots with Bio-Rad ChemiDoc XRS system (Hercules, CA, USA). Protein expression levels were quantified with ImageJ software (NIH, Bethesda, MD, USA).

### Enzyme-Linked Immunosorbent Assay (ELISA)

Cells were seeded in 25 cm^2^ culture flasks at an appropriate density, resulting in 80% confluence within 16-24 hours. When the cell confluence reached 70-80%, rinsed the cell layer with PBS and cultured the cells in 5 mL fresh serum-free medium. Supernatants were harvested 24 hours later and used for subsequent ELISA. The secreted CTGF protein levels in the medium were measured with a Human CTGF ELISA kit. The total protein concentration was also examined, and then the concentrations of secreted protein of different samples were normalized to corresponding total protein (43).

### Lentiviral Transduction

The pLV-EF1α-CTGF-CMV-Puro lentiviral plasmid (CTGF-OE), negative control lentiviral plasmid (NC), CTGF-specific shRNAs (sh-CTGF), negative control shRNAs and the according lentivirus were purchased from Shanghai GeneChem Co. (Shanghai, China). Viral titres of approximately 1 × 10^9^ infectious units/ml were obtained. Lentivirus infection was performed with polybrene according to the manufacturer’s instructions. Briefly, cells were seeded in 12-well plates (3-5 × 10^4^ cells/well) and cultured for 16-24 hours. Then, the culture medium was replaced with 480 μL fresh culture medium and 20μL polybrene (25×) per well. Lentivirus was then added to the culture medium at MOI (multiplicity of infection) of 20 (MDA-MB-231 and HS578T cells) or 10 (HFF1 and WI-38 cells).


MOI=(virus titres×virus volume)/cell counts

Cells were incubated for 16 hours with lentivirus. Then the culture medium containing lentivirus was replaced with 1 mL fresh culture medium per well for the continuous culture. The stably transfected cells were selected by 1 μg/mL puromycin for 7 days. The sequences of CTGF-specific shRNAs are listed in **Table S3**.

### Transwell Migration Assay

Transwell migration assays were performed using a 24-well Transwell chamber with a pore size of 8 µm (Costar, San Diego, CA, USA). Cancer cells (1 × 10^5^) were maintained in serum-free culture medium in the upper chamber. Medium containing 10% FBS was placed in the lower chamber as a chemoattractant. Cancer cells were allowed to migrate for 24 h at 37°C in a humidified atmosphere containing 5% CO_2_. Subsequently, cells that had failed to migrate were removed from the upper chamber with swabs; the remaining cells on the bottom side of the basement membrane were fixed with 4% paraformaldehyde and stained with 1% crystal violet (44). The cells in the lower portion of transwell membrane were counted. A random selection of 3–5 fields were photographed and counted under the microscope (Olympus BX40, Tokyo, Japan).

### Co-Culture Experiments

Co-culture was carried out as described by Su et al. (6). Co-culture experiments were performed using 6-well or 24-well Transwell chambers with a 0.4-µm pore size (Costar, San Diego, CA, USA). Taking 6-well Transwell chambers for example, TNBC cells (1 × 10^5^) were seeded in the lower chamber with 2.5 mL culture medium, and fibroblasts (1 × 10^5^) were seeded in the upper chamber with 1.5 mL culture medium. DMEM supplemented with 10% FBS was used for HS578T and WI-38 cells co-culture experiments. DMEM supplemented with 15% FBS was used for MDA-MB-231 and HFF1 cells co-culture experiments.

### CCK8 Assay

Cell Counting Kit-8 was bought from Beijing Solarbio Science & Technology Co. (Beijing, China). Cells were seeded onto 96-well plates or 24-well plates at a density of 1×10^4^ cells/mL with indicated treatment. Six to eight parallel wells were assigned to each group. At different time points, the culture medium was replaced with 100 μl fresh medium per 96 well or 500 μl fresh medium per 24 well. Then, CCK-8 solution (10 μl per 96 well or 50 μl per 24 well) was added, followed by incubation for 2 h at 37 °C. A multiplate reader (Flexstation^®^ 3, Molecular Devices, LLC., San Jose, CA, USA) was used to measure absorbance at 450 nm.

### Trypan Blue Exclusion Assay

Adherent cells were digested with trypsin into a single-cell suspension. The cell suspension was mixed with a 0.4% trypan blue solution at a ratio of 9:1. The live and dead cells were counted within 3 minutes. The proportion of live cells was then calculated.

### *In Vivo* Assay

MDA-MB-231 cells (6 × 10^6^) were implanted into the fat pads of 6-week-old Balb/c mice. Tumor volumes were measured every three days. Tumor volume was calculated using the formula Volume (mm^3^) = (length × height^2^)/2. When the tumors reached approximately 3 mm in diameter, paclitaxel treatment was started at a dose of 10 mg/kg i.p. once per week. ATL-1 was given at 50 mg/kg i.p. once daily as described by Li Y et al. (18). After 6 weeks of treatment, all the animals were sacrificed. Partial fresh primary tumors were used for primary culture of fibroblasts as described by Calvo et al. (45). Partial primary tumors and mouse organs were fixed in 10% formalin and embedded in paraffin for subsequent analysis.

### Primary Culture of Cancer-Associated Fibroblasts

Primary culture was carried out as described by Calvo et al. (45), with some modifications. Tumor samples were cut into small pieces and digested by collagenase/dispase. Then, 44 μm nylon meshes were used to remove undigested tissue. After centrifuging at 1000 rpm/min for 10 min, the filtered cells were collected and then re-suspended in DMEM with 10% FBS and cultured in the culture dish. Thirty minutes later, the fibroblasts had already adhered to the culture dish, while other cell types remained in suspension (45). Afterward, the culture media were changed and fibroblasts were cultured for subsequent experiments.

### Statistical Analysis

Data were assessed using SPSS version 20.0 (SPSS Inc., Chicago, IL, USA). Adobe Illustrator CC 2018 and GraphPad Prism 5.0 (GraphPad Software Inc., San Diego, CA, USA) were used to represent the data. Student’s t test was used to determine the difference between each two groups. Error bars in the experiments indicate standard deviation (SD). Any values of P < 0.05 were considered statistically significant.

## Results

### ATL-1 Inhibited Tumor Cell Migration and Increased the Sensitivity of Tumor Cells to Paclitaxel

**Figure 1:**
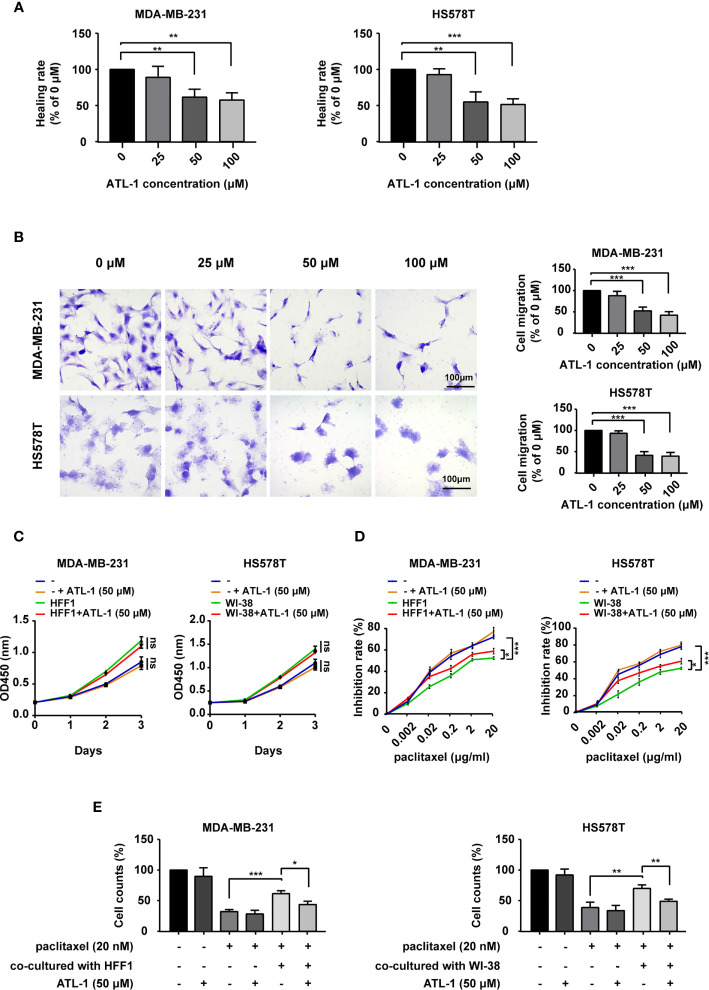
ATL-1 inhibited tumor cell migration and increased the sensitivity of tumor cells to paclitaxel. Wound healing **(A)** and Transwell migration **(B)** assays showed that 50 μM and 100 μM ATL-1 reduced the migratory capacities of MDA-MB-231 and HS578T cells. **(C)** CCK8 assays showed that 50 μM ATL-1 alone had no growth-inhibiting effect on MDA-MB-231 and HS578T cells cultured alone (–) or co-cultured with fibroblasts (HFF1 or WI-38 cells). **(D)** CCK8 assays showed the growth-inhibiting effects of paclitaxel on MDA-MB-231 and HS578T cells cultured alone (–) or co-cultured with fibroblasts (HFF1 or WI-38 cells) with or without 50 μM ATL-1 treatment for 48 hrs. **(E)** Trypan blue exclusion assay showed the live cell counts after paclitaxel treatment on MDA-MB-231 and HS578T cells cultured alone or co-cultured with fibroblasts with or without 50 μM ATL-1 treatment for 48 hrs. **(A–E)** Three technical replicates were performed for each of the three biological replicates. Mean ± SD, *p < 0.05, **p < 0.01, ***p < 0.001 by Student’s t test.

As shown in **Figures 1A, B**, we found that 50 μM and 100 μM ATL-1 significantly inhibited the migration of MDA-MB-231 and HS578T TNBC cells in wound healing assays and Transwell migration assays. We chose 50μM as the ATL-1 concentration for subsequent experiments. To mimic the tumor microenvironment, we co-cultured TNBC cells and fibroblasts (HFF1 and WI38 cells), and then examined the growth effects of ATL-1 on MDA-MB-231 and HS578T cells by CCK8 assays. We found 50 μM ATL-1 alone had no growth-inhibiting effect on TNBC cells cultured alone (–) or co-cultured with fibroblasts (**Figures 1C**, **S1A**). We then examined the sensitivity of the tumor cells to paclitaxel by CCK8 assays and trypan blue exclusion assays. We found that when TNBC cells were challenged with paclitaxel for 48 hrs, the inhibition rate of TNBC cells in co-cultured systems was lower than that of TNBC cells cultured alone (-)(**Figures 1D**, **S1B**), and the proportion of survived TNBC cells in co-cultured systems was higher than that of TNBC cells cultured alone (**Figures 1E**, **S1C**). However, we found that when TNBC cells were challenged with paclitaxel in co-cultured systems, the inhibition rate of TNBC cells with 50 μM ATL-1 treatment was higher than that of TNBC cells without ATL-1 treatment (**Figures 1D**, **S1B**), and the proportion of survived TNBC cells with ATL-1 treatment was lower than that of TNBC cells without ATL-1 treatment (**Figures 1E**, **S1C**). These data suggested that ATL-1 inhibited tumor cell migration and increased the inhibitory effects of paclitaxel on TNBC cells in co-culture systems.

### ATL-1 Downregulates CTGF Expression in Triple-Negative Breast Cancer Cells and Inhibits Cancer Cell Migration *via* CTGF

**Figure 2:**
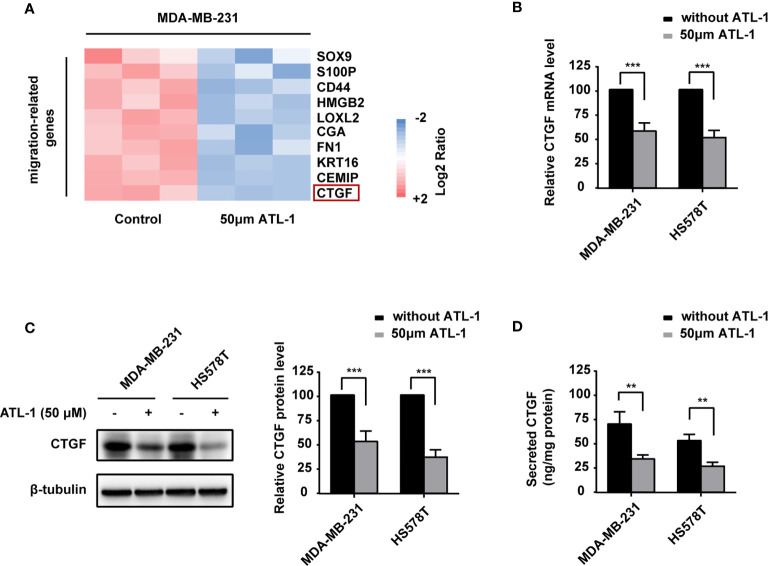
ATL-1 downregulates CTGF expression in triple-negative breast cancer cells and inhibits cancer cell migration *via* CTGF. **(A)** Heatmaps of migration-related gene-expressions from MDA-MB-231 qRT-PCR analysis. MDA-MB-231 cells were treated with/without 50 μM ATL-1 for 24 h. Pink and blue colors represent gene expression levels above and below the mean, respectively. **(B)** qPCR illustrated the downregulation of CTGF mRNA in MDA-MB-231 and HS578T cells after treatment with 50 μM ATL-1 for 24 hrs. Western blotting **(C)** and ELISA **(D)** illustrated downregulation of the CTGF protein and secreted protein levels of MDA-MB-231 and HS578T cells after treatment with 50 μM ATL-1 for 24 hrs. **(A–D)** Three technical replicates were performed for each of the three biological replicates. Mean ± SD, **p < 0.01, ***p < 0.001 compared to without ATL-1 treatment by Student’s t test.

To reveal the potential mechanism by which ATL-1 inhibits TNBC cell migration, we examined differentially expressed migration-related genes in MDA-MB-231 TNBC cells by qRT-PCR after ATL-1 treatment. The normalized log2 ratio and p value for significantly downregulated genes were shown in **Table S1**. We found that the CTGF mRNA level was significantly decreased after treatment with 50 μM ATL-1 for 24 hrs (**Figure 2A**). qRT-PCR and Western blotting verified the downregulation of CTGF at the mRNA and protein levels in MDA-MB-231 and HS578T TNBC cells following ATL-1 treatment (**Figures 2B, C**). ELISA verified the downregulation of the secreted level of CTGF (**Figure 2D**).

### ATL-1 Inhibits Triple-Negative Breast Cancer Cell Migration *via* CTGF

**Figure 3:**
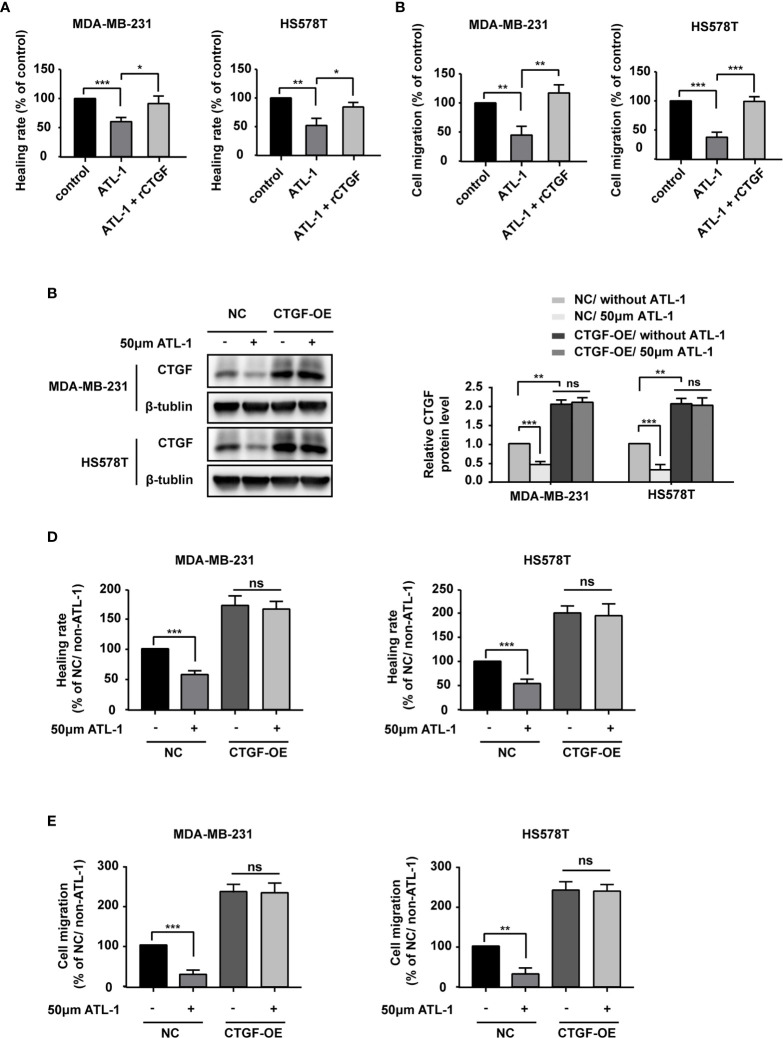
ATL-1 inhibits triple-negative breast cancer cell migration *via* CTGF. Wound healing **(A)** and transwell migration **(B)** assays showed that the reductions in MDA-MB-231 and HS578T cell migration induced by ATL-1 were rescued by rCTGF. **(C)** Western blotting demonstrated that CTGF was overexpressed in MDA-MB-231 and HS578T cells by CTGF-OE lentivirus. After being overexpressed, there was no difference in CTGF expression between with and without ATL-1 treatment. Wound healing **(D)** and Transwell migration **(E)** assays showed that the reductions in MDA-MB-231 and HS578T cell migration induced by ATL-1 were attenuated in CTGF-OE cells. **(A–E)** Three technical replicates were performed for each of the three biological replicates. Mean ± SD, *p < 0.05, **p < 0.01, ***p < 0.001 by Student’s t test.

We wondered whether ATL-1 inhibits TNBC cell migration *via* CTGF. We found that recombinant CTGF (rCTGF) could rescue the downregulation of cancer cell migration by ATL-1 in wound healing assays and Transwell migration assays (**Figures 3A, B**). To further determine the role of CTGF, we overexpressed the expression of CTGF with CTGF-OE lentivirus in MDA-MB-231 and HS578T cells (CTGF-OE), and used the NC lentivirus as the negative control (NC) (**Figure 3C**). After being overexpressed, there was no difference in CTGF expression between with and without ATL-1 treatment (**Figure 3C**). We found that the reduction in migration induced by ATL-1 could be attenuated in CTGF-OE cells (**Figures 3D, E**). We also knocked down the expression of CTGF with shRNAs in MDA-MB-231 and HS578T cells (**Figure S2A**). We found that the reduction in migration induced by ATL-1 could be attenuated by CTGF-specific shRNAs (**Figures S2B, C**). These data further confirmed that CTGF mediated the inhibitory effect of ATL-1 on tumor cell migration.

### ATL-1 Downregulates Fibroblast Expression of CTGF and Inhibits the Ability of Breast Cancer Cells to Transform Fibroblasts Into CAF-Like Cells

**Figure 4:**
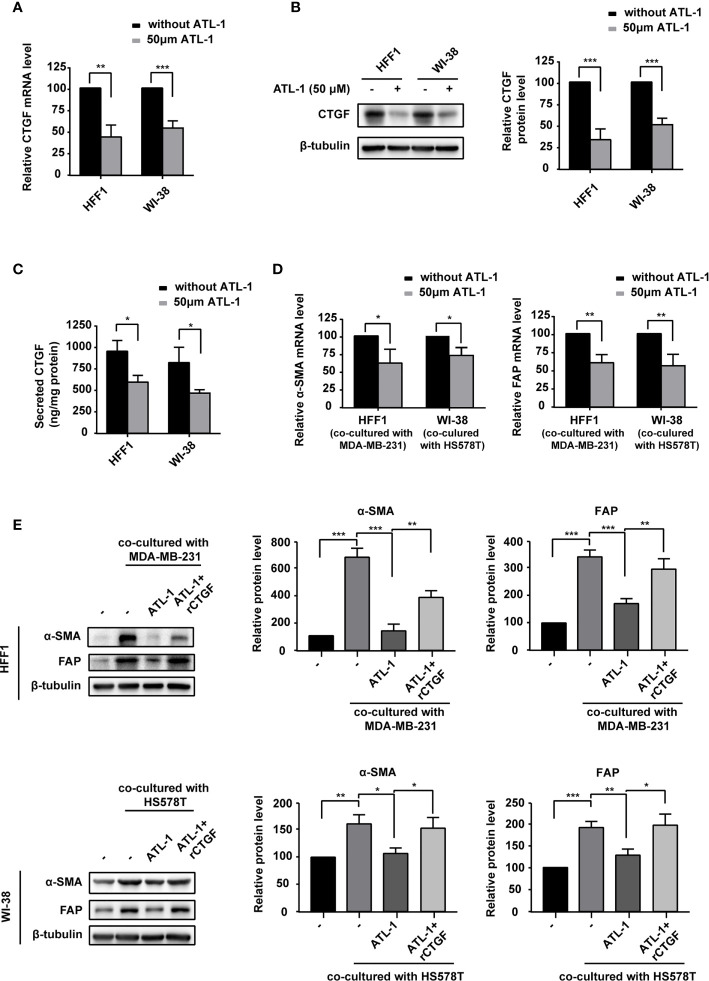
ATL-1 downregulates fibroblast expression of CTGF and inhibits the ability of breast cancer cells to transform fibroblasts into CAF-like cells. **(A)** qPCR illustrated downregulation of CTGF mRNA in HFF1 and WI-38 cells after treatment with 50 μM ATL-1 for 24 hrs. Western blotting **(B)** and ELISA **(C)** illustrated downregulation of the CTGF protein and secreted protein levels of HFF1 and WI-38 cells after treatment with 50 μM ATL-1 for 24 hrs. qRT-PCR **(D)** demonstrated that treatment with 50 μM ATL-1 in the co-culture system for 48 hrs downregulated the mRNA levels of CAF markers compared with no ATL-1 treatment. Western blotting **(E)** demonstrated that co-culture with cancer cells upregulated the protein levels of the CAF markers FAP and α-SMA in HFF1 and WI-38 cells. Treatment with 50 μM ATL-1 in the co-culture system for 48 hrs downregulated the protein levels of CAF markers compared with no ATL-1 treatment. rCTGF rescued the downregulation. (-) in **(E)** represent without ATL-1 and rCTGF treatment. **(A–E)** Three technical replicates were performed for each of the three biological replicates. Mean ± SD, *p < 0.05, **p < 0.01, ***p < 0.001 by Student’s t test.

Because CTGF is associated with the transformation of fibroblasts into CAFs and chemotherapy resistance (35), we examined the effect of ATL-1 on CTGF expression in fibroblasts. We found that CTGF mRNA and protein levels were significantly decreased by ATL-1 treatment in HFF1 and WI-38 fibroblasts (**Figures 4A, B**). ELISA verified the downregulation of the secreted level of CTGF (**Figure 4C**). To mimic fibroblast transformation in the tumor microenvironment, we co-cultured TNBC cells and fibroblasts for 2 days and then examined the mRNA and protein levels of the CAF markers FAP and α-SMA. Firstly, we examined the effect of recombinant CTGF (rCTGF) on the transformation of fibroblasts into CAFs in co-culture systems. We found that the treatment with rCTGF (higher than 5 μg/mL) for 2 days could significantly increase the mRNA levels of FAP and α-SMA in co-cultured systems, and we chose 10 μg/mL for the subsequence experiments (**Supplementary Figures S3A, B**). Then, we examined the effects of ATL-1 on fibroblasts transformation. We found that treatment with 50 μM ATL-1 for 2 days dramatically reduced the mRNA and protein levels of FAP and α-SMA in co-cultured systems, which could be rescued by rCTGF (**Figures 4D, E**; **Supplementary Figure S3C**).

### ATL-1 Increased the Sensitivity of TNBC Cells to Paclitaxel by Downregulating the Expression of CTGF in Fibroblasts

**Figure 5:**
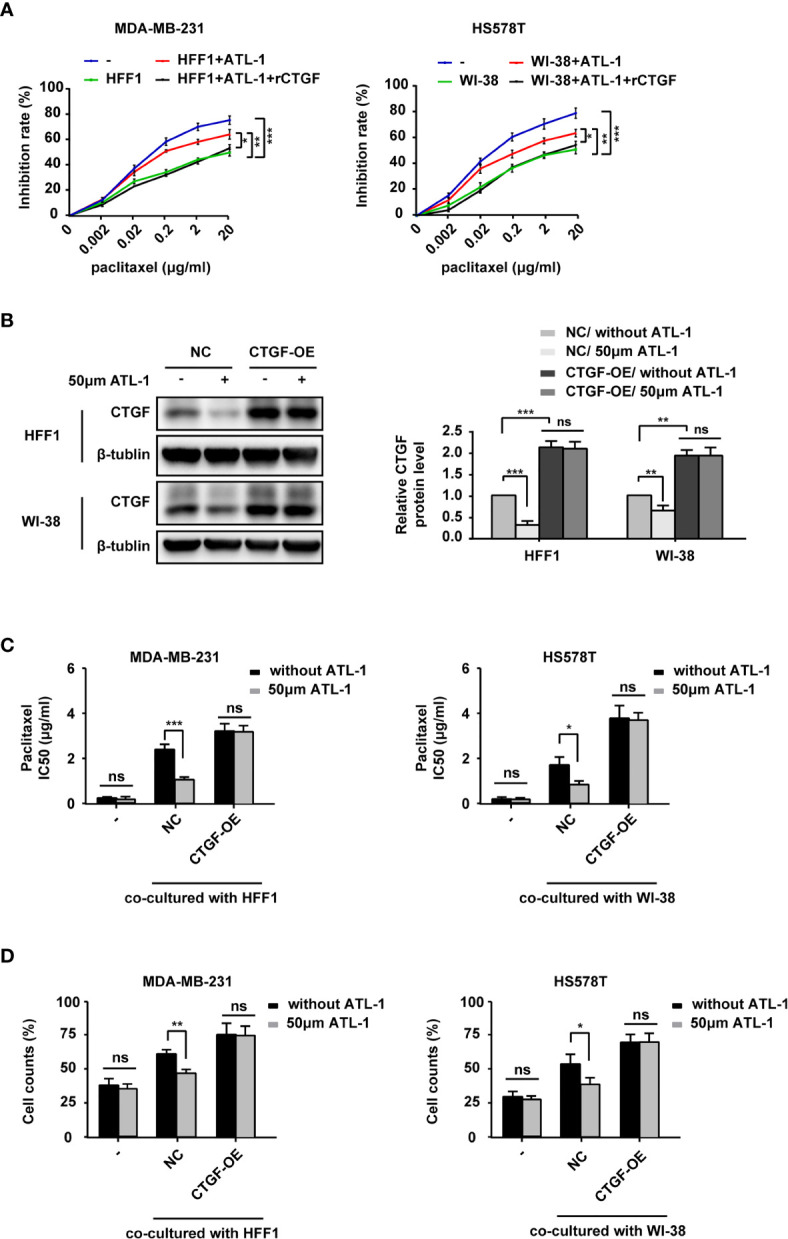
ATL-1 increased the sensitivity of TNBC cells to paclitaxel by downregulating the expression of CTGF in fibroblasts. CCK8 **(A)** assay showed the growth-inhibiting effects of paclitaxel on MDA-MB-231 and HS578T cells cultured alone (–) or co-cultured with fibroblasts with or without ATL-1 treatment and rCTGF treatment for 48 hrs. **(B)** Western blotting demonstrated that CTGF expression was overexpressed in HFF1 and WI-38 cells. After being overexpressed, there was no difference in CTGF expression between with and without ATL-1 treatment. **(C)** CCK8 showed the IC50 of paclitaxel on MDA-MB-231 and HS578T cells cultured alone (–) or co-cultured with fibroblasts with or without ATL-1 treatment for 48 hrs. **(D)** Trypan blue exclusion assay showed the live cell counts after paclitaxel treatment on MDA-MB-231 and HS578T cells cultured alone(–) or co-cultured with fibroblasts with or without 50 μM ATL-1 treatment for 48 hrs. ATL-1 increased the growth-inhibiting effects of paclitaxel in co-culture systems (NC), which was attenuated by the overexpression of CTGF in fibroblasts (CTGF-OE). **(A–D)** Three technical replicates were performed for each of the three biological replicates. Mean ± SD, *p < 0.05, **p < 0.01, ***p < 0.001 by Student’s t test.

To study the role of CTGF in the sensitization of TNBC cells to chemotherapy, we co-cultured TNBC cells and fibroblasts and then examined the effects of ATL-1 and rCTGF on the sensitivity of the tumor cells to paclitaxel by CCK8 assays. All treatments lasted 48 hrs. We found that the addition of ATL-1 could obviously increase the inhibition rates of TNBC cells in the co-culture systems, which could be attenuated by rCTGF (**Figures 5A**, **S4A**). We then overexpressed CTGF expression with CTGF-OE lentivirus in HFF1 and WI-38 fibroblasts (CTGF-OE), and used the NC lentivirus as the negative control (NC) (**Figure 5B**). After being overexpressed, there was no difference in CTGF expression between with and without ATL-1 treatment (**Figure 5B**). We examined the sensitivity of tumor cells to paclitaxel with CCK8 assays and trypan blue exclusion assays. All treatments lasted 48 hrs. We found that when TNBC cells were co-cultured with NC fibroblasts, the IC50 of paclitaxel and the proportion of survived TNBC cells after paclitaxel treatment were reduced by ATL-1 treatment. While, when TNBC cells were co-cultured with CTGF-OE fibroblasts, the IC50 of paclitaxel and the proportion of survived TNBC cells after paclitaxel treatment were no longer reduced by ATL-1 treatment (**Figures 5C, D**; **Figures S4B, C**). We also knocked down CTGF expression with shRNAs in HFF1 and WI-38 fibroblasts (**Figure S5A**) and found that the ATL-1-driven chemosensitization in co-cultured systems could be attenuated by CTGF-specific shRNAs acting on fibroblasts (**Figures S5B, C**). These data suggest that CTGF secreted by fibroblasts mediates the chemosensitizing effect of ATL-1 on TNBC cells.

### Atractylenolide-I Sensitizes Triple-Negative Breast Cancer to Paclitaxel in a Mouse Model

**Figure 6:**
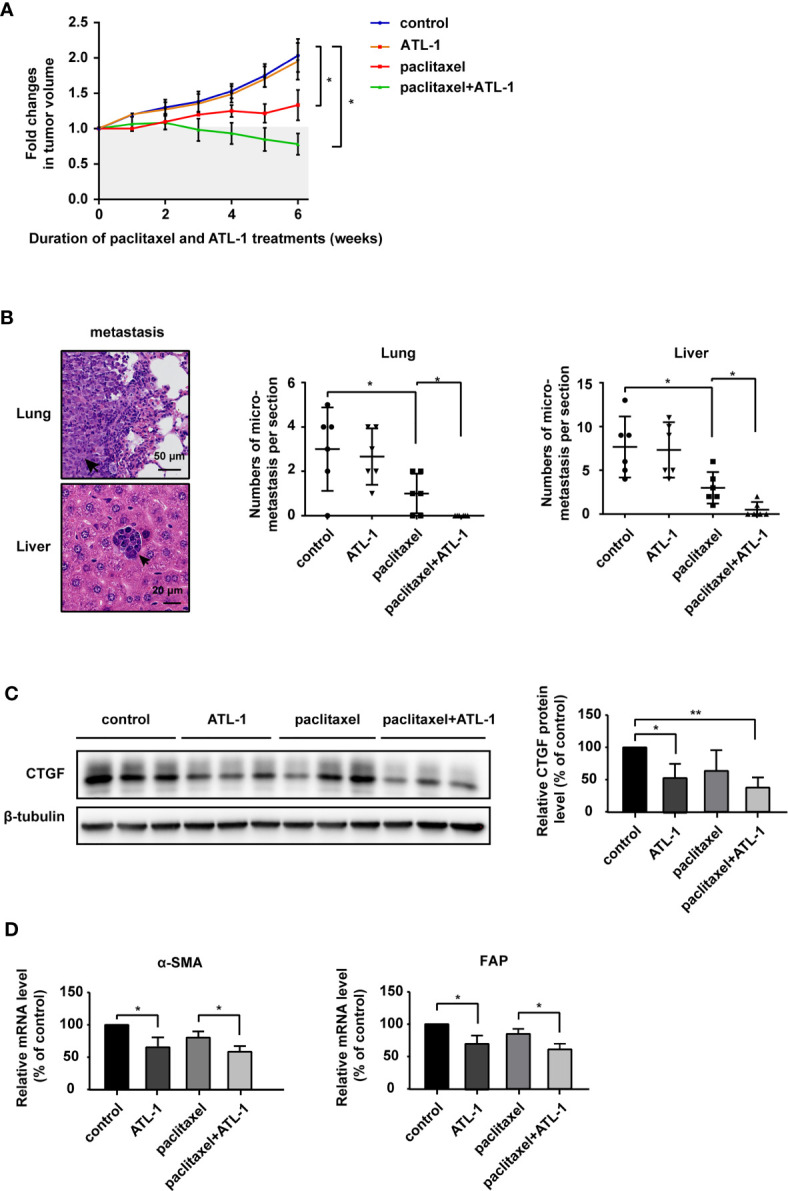
Atractylenolide-I sensitizes triple-negative breast cancer to paclitaxel in a mouse model. **(A)** MDA-MB-231 cells were inoculated orthotopically into the mammary fat pad of 6-week-old female Balb/c mice (n = 6). When the tumors reached approximately 3 mm in diameter, paclitaxel treatment and ATL-1 treatment were started. Primary tumor size was measured and quantified every three days for 6 weeks. The abscissa represents the time after the start of treatment. **(B)** Representative images of metastatic tumors in lungs and liver, which were stained with HE. The numbers of micrometastatic lesions in the lungs and liver were quantified. **(C)** The expression of CTGF in primary tumors was examined by Western blotting. **(D)** qRT-PCR was performed to evaluate the mRNA levels of the CAF markers FAP and α-SMA in primary cultured fibroblasts derived from inoculated tumors. **(A, B)** Mean ± SD, *p < 0.05 by Student’s t test. **(C, D)** Three independent experiments were performed for each of the Balb/c mice in the four groups. Mean ± SD, *p < 0.05, **p < 0.01 by Student’s t test.

Finally, we analyzed the effect of ATL-1 on chemosensitivity *in vivo*. We found that in model mice bearing subcutaneously inoculated MDA-MB-231 cells, paclitaxel treatment inhibited tumor growth and lung and liver micrometastases. ATL-1 dramatically enhanced these inhibitory effects (**Figures 6A, B**). However, ATL-1 alone had no effect on tumor growth and lung and liver micrometastases (**Figures 6A, B**). Western blotting showed that ATL-1 could reduce CTGF protein levels in primary tumors (**Figure 6C**). In addition, qRT-PCR revealed that primary cultured fibroblasts derived from tumors treated with ATL-1 alone (ATL-1) expressed lower mRNA levels of FAP and α-SMA than fibroblasts from control tumors (control) (**Figure 6D**). And primary cultured fibroblasts derived from tumors treated with ATL-1 in combination with paclitaxel (paclitaxel+ATL-1) expressed lower mRNA levels of FAP and α-SMA than fibroblasts from tumors treated with paclitaxel alone (paclitaxel) (**Figure 6D**).

## Discussion

Atractylodolactone-1 has been shown to significantly inhibit the tumorigenesis and development of a variety of tumors and increase the sensitivity of tumors to chemotherapy. Long F et al. found that ATL-1 could suppress tumorigenesis in breast cancer by inhibiting the Toll-like receptor 4-mediated NF-κB signaling pathway (17). ATL-1 was found to inhibit melanoma and colorectal cancer cell proliferation *via* the JAK2/STAT3 or AKT/mTOR signaling pathway (18, 20). Ye Y et al. found that ATL-1 could induce apoptosis and cell cycle arrest in melanoma cells *via* ERK/GSK3beta signaling. Ma L et al. reported that ATL-1 could attenuate gastric cancer stem cell traits *via* the Notch pathway (23). However, the effect of ATL-1 on tumor microenvironment has not been studied.

We found that ATL-1 could significantly increase the sensitivity to paclitaxel in triple-negative breast cancer in co-cultured systems. Huang JM et al. found that ATL-1 sensitized human ovarian cancer cells to paclitaxel by blocking activation of the TLR4/MyD88-dependent pathway in cancer cells (22). However, we found that when TNBC cells were cultured alone, ATL-1 was not enough to affect the sensitivity to paclitaxel in TNBC cells. This may be due to the difference in cell types. We are the first to find that ATL-1 could affect chemotherapy sensitivity through fibroblasts in the tumor microenvironment. We found that ATL-1 could inhibit fibroblast transformation into CAFs. Guo Y et al. reported that ATL-1 could repress the myofibroblastic phenotype and fibrosis development in unilateral ureteral obstruction kidneys by targeting fibroblast-myofibroblast differentiation (FMD), as well as epithelial-mesenchymal transition (EMT), which supports our findings (16). We found the chemosensitization function of ATL-1 is inseparable from co-cultured with fibroblasts. We speculate that the addition of ATL-1 could downregulate the expression and secretion levels of CTGF, which could inhibit fibroblasts transform to CAF. The change in fibroblasts’ transformation level could alter some proteins or exosomes secreted by fibroblasts, which in turn affects the sensitivity of tumor cells to paclitaxel. The verification of this hypothesis needs further experiments. It has been reported that CAFs could induce chemoresistance by secreting IL-6, IL-8 (6), HGF (5) and exosomes (7, 8), which explains our findings supporting the conclusion that ATL-1 could inhibit chemotherapy resistance *via* fibroblasts.

To explain the biological function of ATL-1 we have discovered, we examined differentially expressed migration-related genes in MDA-MB-231 cells by qRT-PCR after ATL-1 treatment. Firstly we examined the genes that were involved in our past or on-going experiments. As shown in **Figure 2A**, most of these genes are classic genes closely related to cell migration. The down-regulation of these classic genes by ATL-1 can prove our observation to a certain extent that ATL-1 inhibits cell migration. In addition, most of these classic genes are associated not only with tumor cell migration but also with chemoresistance of tumor cells. They promote chemoresistance by different mechanisms. Because we are going to study the mechanism of ATL-1 on chemosensitization, the examination of these genes can provide ideas for us. For example, SOX9 is a key transcription factor and related with chemoresistance (46); HMGB2 is known to bind to DNA structure resulting from cisplatin-DNA adducts and affect the chemosensitivity of cells (47); CD44 is related with stemness of cancer cells (48); S100A4 and CTGF are related with fibroblast in tumor microenvironment (43, 49). We chose CTGF for further study because it might help us to explain the chemosensitization function of ATL-1 from aspect of microenvironment and CAF. The effect of ATL-1 on tumor microenvironment has not been studied. As ATL-1 was reported to inhibit fibroblast-myofibroblast differentiation (FMD) and repress fibrosis development in unilateral ureteral obstruction kidneys (16). We wondered whether ATL-1 could inhibit fibroblast activation *via* CTGF in tumor microenvironment. As CTGF and CAF in tumor microenvironment have successfully explained the chemosensitization function of ATL-1 in the following experiments, we did not further examine other genes by RNA-seq at that moment.

We found that the expression of CTGF in TNBC cells and fibroblasts could be reduced by ATL-1, which led to a decrease in cancer cell migration and an increase in chemosensitivity. Several studies have reported that CTGF can promote the migration of tumor cells (50–52), which supports our view. Our data also showed that CTGF expression was significantly higher in fibroblasts than in tumor cells (**Figures 2D**, **4C**). This also explains, to some extent, why ATL-1 treatment in co-culture systems could significantly increase chemotherapy sensitivity compared with treatment in single-culture systems of tumor cells. As for how the significant reduction of CTGF secretion level in fibroblasts affected the chemosensitivity of TNBC cells, we have the following two hypotheses. On the one hand, ATL-1 can directly affect the sensitivity of tumor cells to chemotherapy by reducing the secretion of CTGF by fibroblasts. CTGF has been reported to increase drug resistance to paclitaxel by upregulating survivin expression in human osteosarcoma cells (53). CTGF can promote chemoresistance in glioblastoma through TGF-β1-dependent activation of Smad/ERK signaling (54). Even *in vivo* experiments have shown that CTGF antagonism with the mAb FG-3019 enhances the chemotherapy response in murine models of pancreatic ductal adenocarcinoma (55). On the other hand, but to some extent that we think is more important, CTGF can promote the transformation of fibroblasts into CAFs, and CAFs can promote chemotherapy resistance. ATL-1 can inhibit the transformation of fibroblasts into CAFs induced by CTGF and indirectly increase the chemotherapy sensitivity of tumor cells in a co-culture system. CTGF can induce a variety of cells to transform into CAFs in the tumor microenvironment. Tsang M et al. found that CTGF is required for the activation of cancer-associated fibroblasts in a murine model of melanoma (56). As a downstream effector of the profibrotic molecule TGF-β, CTGF can promote the differentiation of hepatic stellate cells into tumor-promoting myofibroblasts (33). All these observations support our findings.

CTGF expression was reported to be controlled by TGFB1 signaling in fibroblasts and Hippo-YAP signaling in TNBC cells (57–59). In our results, we found that CTGF expression was reduced by ATL-1 in TNBC cells and fibroblasts. But the specific activated receptors and signaling pathways need to be further studied. RNA-seq, KEGG and GO analysis will be need for our further research.

Our mice model showed that paclitaxel treatment and paclitaxel combined with ATL-1 treatment could reduce tumor volume and numbers of metastasis. Compared with single paclitaxel treatment, the addition of ATL-1 treatment could further enhance therapeutic effect. This was consistent with our results *in vitro*. But, we did not see any effect on single ATL-1 treatment on metastasis, though we saw ATL-1 treatment could inhibit TNBC cells migration *via* CTGF in the in-vitro model. Actually, metastasis is facilitated by at least four essential steps: detachment, migration, invasion and adhesion (60). These four essential, metastatic steps are inter-related and affected by multi-biochemical events and parameters (60). Just reducing the migration ability by ATL-1 may not be sufficient to reduce tumor metastasis *in vivo* models. Paclitaxel can cause the death of tumor cells by disrupting the normal microtubule dynamics required for cell division and vital interphase processes (61). Paclitaxel can significantly inhibit the growth of primary tumors and metastatic tumors and has been widely used in clinical practice. Although the reduction of CTGF and fibroblasts transformation induced by single ATL-1 treatment were not sufficient to inhibit tumor metastasis, these two steps played key roles in increasing the sensitivity of paclitaxel chemotherapy, and we have seen the enhanced therapeutic effect of the combination therapy in our *in vivo* models.

In summary, our study demonstrated that ATL-1 could inhibit tumor cell migration *via* downregulation of CTGF in triple-negative breast cancer cells. Moreover, ATL-1 reduced the expression of CTGF in fibroblasts and inhibited fibroblast transformation into CAFs. ATL-1 increased the sensitivity of TNBC cells to paclitaxel by downregulating the expression of CTGF in fibroblasts. These results indicate that ATL-1 can increase chemosensitivity to paclitaxel and suppress breast cancer metastasis. Our findings provide a theoretical basis for the clinical application of ATL-1.

## Data Availability Statement

The original contributions presented in the study are included in the article/**Supplementary Material**. Further inquiries can be directed to the corresponding author.

## Ethics Statement

The animal study was reviewed and approved by Ethics Committee of Panyu Hospital of Chinese Medicine (No. 2019011).

## Author Contributions

XC designed the studies and wrote the manuscript. MW performed the experiments, analyzed data, and wrote the manuscript. X-ZL, M-XZ, and Q-YY helped with the experiments. Y-XC and XC suggested experiments and revised the manuscript. All authors contributed to the article and approved the submitted version.

## Funding

This work was supported by grants to XC. from the Scientific Research Project of Traditional Chinese Medicine Bureau of Guangdong Province of China (No. 20201280).

## Conflict of Interest

The authors declare that the research was conducted in the absence of any commercial or financial relationships that could be construed as a potential conflict of interest.

## Publisher’s Note

All claims expressed in this article are solely those of the authors and do not necessarily represent those of their affiliated organizations, or those of the publisher, the editors and the reviewers. Any product that may be evaluated in this article, or claim that may be made by its manufacturer, is not guaranteed or endorsed by the publisher.

## Supplementary Material

Supplementary Figure 1 ATL-1 increased the sensitivity of tumor cells to paclitaxel. **(A)** CCK8 assays showed that 50 μM ATL-1 alone had no growth-inhibiting effect on MDA-MB-231 and HS578T cells cultured alone (–) or co-cultured with fibroblasts (HFF1 or WI-38 cells). **(B)** CCK8 assays showed the growth-inhibiting effects of paclitaxel on MDA-MB-231 and HS578T cells cultured alone (–) or co-cultured with fibroblasts (HFF1 or WI-38 cells) with or without 50 μM ATL-1 treatment for 48 hrs. **(C)** Trypan blue exclusion assay showed the live cell counts after paclitaxel treatment on MDA-MB-231 and HS578T cells cultured alone or co-cultured with fibroblasts with or without 50 μM ATL-1 treatment for 48 hrs. **(A–C)** Three technical replicates were performed for each of the three biological replicates. Mean ± SD, *p < 0.05, **p < 0.01, ***p < 0.001 by Student’s t test.

Supplementary Figure 2 ATL-1 inhibits triple-negative breast cancer cell migration *via* CTGF. **(A)** Western blotting demonstrated that CTGF expression was knocked down in MDA-MB-231 and HS578T cells. Wound healing **(B)** and transwell migration **(C)** assays showed that the reductions in MDA-MB-231 and HS578T cell migration induced by ATL-1 were attenuated by sh-CTGF. Three technical replicates were performed for each of the three biological replicates. Mean ± SD, **p < 0.01, ***p < 0.001 by Student’s t test.

Supplementary Figure 3 The effects of recombinant CTGF (rCTGF) and ATL-1 on the transformation of fibroblasts into CAFs in co-culture systems. **(A, B)** qRT-PCR demonstrated that treatment with different concentrate of rCTGF in the co-culture system for 48 hrs had different effect on the mRNA levels of CAF markers (FAP and α-SMA) compared with no rCTGF treatment. **(C)** qRT-PCR demonstrated that treatment with 50 μM ATL-1 in the co-culture system for 48 hrs downregulated the mRNA levels of CAF markers compared with no ATL-1 treatment. Three technical replicates were performed for each of the three biological replicates. Mean ± SD, *p < 0.05, **p < 0.01, ***p < 0.001 by Student’s t test.

Supplementary Figure 4 ATL-1 increased the sensitivity of TNBC cells to paclitaxel by downregulating the expression of CTGF in fibroblasts. CCK8 **(A)** assay showed the growth-inhibiting effects of paclitaxel on MDA-MB-231 and HS578T cells cultured alone (–) or co-cultured with fibroblasts with or without ATL-1 treatment and rCTGF treatment for 48 hrs. **(B)** CCK8 showed the IC50 of paclitaxel on MDA-MB-231 and HS578T cells cultured alone (–) or co-cultured with fibroblasts with or without ATL-1 treatment for 48 hrs. **(C)** Trypan blue exclusion assay showed the live cell counts after paclitaxel treatment on MDA-MB-231 and HS578T cells cultured alone(–) or co-cultured with fibroblasts with or without 50 μM ATL-1 treatment for 48 hrs. ATL-1 increased the growth-inhibiting effects of paclitaxel in co-culture systems (NC), which was attenuated by the overexpression of CTGF in fibroblasts (CTGF-OE). **(A–C)** Three technical replicates were performed for each of the three biological replicates. Mean ± SD, *p < 0.05, **p < 0.01, ***p < 0.001 by Student’s t test.

Supplementary Figure 5 ATL-1 increased the sensitivity of TNBC cells to paclitaxel by downregulating the expression of CTGF in fibroblasts. **(A)** Western blotting demonstrated that CTGF expression was knocked down in HFF1 and WI-38 cells. **(B)** CCK8 showed the IC50 of paclitaxel on MDA-MB-231 and HS578T cells cultured alone (–) or co-cultured with fibroblasts with or without ATL-1 treatment. **(C)** Trypan blue exclusion assay showed the cell counts after paclitaxel treatment on MDA-MB-231 and HS578T cells cultured alone (-) or co-cultured with fibroblasts with or without 50 μM ATL-1 treatment. ATL-1 increased the growth-inhibiting effects of paclitaxel in co-culture systems (NC), which was attenuated by sh-CTGF. **(A–C)** Three technical replicates were performed for each of the three biological replicates. Mean ± SD, *p < 0.05, **p < 0.01, ***p < 0.001 by Student’s t test.

## References

[1] Loibl S Poortmans P Morrow M Denkert C Curigliano G. Breast Cancer. Lancet (2021) 397(10286):1750–69. doi: 10.1016/S0140-6736(20)32381-3 33812473

[2] Wu Q Yang Z Nie Y Shi Y Fan D. Multi-Drug Resistance in Cancer Chemotherapeutics: Mechanisms and Lab Approaches. Cancer Lett (2014) 347(2):159–66. doi: 10.1016/j.canlet.2014.03.013 24657660

[3] Ganguly D Chandra R Karalis J Teke M Aguilera T Maddipati R. Cancer-Associated Fibroblasts: Versatile Players in the Tumor Microenvironment. Cancers (Basel) (2020) 12(9):2652. doi: 10.3390/cancers12092652 PMC7564346 32957515

[4] Shintani Y Fujiwara A Kimura T Kawamura T Funaki S Minami M. IL-6 Secreted From Cancer-Associated Fibroblasts Mediates Chemoresistance in NSCLC by Increasing Epithelial-Mesenchymal Transition Signaling. J Thorac Oncol (2016) 11(9):1482–92. doi: 10.1016/j.jtho.2016.05.025 27287412

[5] Straussman R Morikawa T Shee K Barzily-Rokni M Qian ZR Du J. Tumour Micro-Environment Elicits Innate Resistance to RAF Inhibitors Through HGF Secretion. Nature (2012) 487(7408):500–4. doi: 10.1038/nature11183 PMC3711467 22763439

[6] Su S Chen J Yao H Liu J Yu S Lao L. CD10(+)GPR77(+) Cancer-Associated Fibroblasts Promote Cancer Formation and Chemoresistance by Sustaining Cancer Stemness. Cell (2018) 172(4):841–56.e16. doi: 10.1016/j.cell.2018.01.009 29395328

[7] Richards KE Zeleniak AE Fishel ML Wu J Littlepage LE Hill R. Cancer-Associated Fibroblast Exosomes Regulate Survival and Proliferation of Pancreatic Cancer Cells. Oncogene (2017) 36(13):1770–8. doi: 10.1038/onc.2016.353 PMC5366272 27669441

[8] Ren J Ding L Zhang D Shi G Xu Q Shen S. Carcinoma-Associated Fibroblasts Promote the Stemness and Chemoresistance of Colorectal Cancer by Transferring Exosomal lncRNA H19. Theranostics (2018) 8(14):3932–48. doi: 10.7150/thno.25541 PMC6071523 30083271

[9] Leung CS Yeung TL Yip KP Wong KK Ho SY Mangala LS. Cancer-Associated Fibroblasts Regulate Endothelial Adhesion Protein LPP to Promote Ovarian Cancer Chemoresistance. J Clin Invest (2018) 128(2):589–606. doi: 10.1172/JCI95200 29251630 PMC5785271

[10] Hingorani SR Zheng L Bullock AJ Seery TE Harris WP Sigal DS. HALO 202: Randomized Phase II Study of PEGPH20 Plus Nab-Paclitaxel/Gemcitabine *Versus* Nab-Paclitaxel/Gemcitabine in Patients With Untreated, Metastatic Pancreatic Ductal Adenocarcinoma. J Clin Oncol (2018) 36(4):359–66. doi: 10.1200/JCO.2017.74.9564 29232172

[11] Olive KP Jacobetz MA Davidson CJ Gopinathan A McIntyre D Honess D. Inhibition of Hedgehog Signaling Enhances Delivery of Chemotherapy in a Mouse Model of Pancreatic Cancer. Science (2009) 324(5933):1457–61. doi: 10.1126/science.1171362 PMC2998180 19460966

[12] Endo K Taguchi T Taguchi F Hikino H Yamahara J Fujimura H. Antiinflammatory Principles of Atractylodes Rhizomes. Chem Pharm Bull (Tokyo) (1979) 27(12):2954–8. doi: 10.1248/cpb.27.2954 540333

[13] Wang HX Liu CM Liu Q Gao K. Three Types of Sesquiterpenes From Rhizomes of Atractylodes Lancea. Phytochemistry (2008) 69(10):2088–94. doi: 10.1016/j.phytochem.2008.04.008 18511090

[14] Li W Zhi W Liu F He Z Wang X Niu X. Atractylenolide I Restores HO-1 Expression and Inhibits Ox-LDL-Induced VSMCs Proliferation, Migration and Inflammatory Responses In Vitro. Exp Cell Res (2017) 353(1):26–34. doi: 10.1016/j.yexcr.2017.02.040 28274716

[15] Zhang JL Huang WM Zeng QY. Atractylenolide I Protects Mice From Lipopolysaccharide-Induced Acute Lung Injury. Eur J Pharmacol (2015) 765:94–9. doi: 10.1016/j.ejphar.2015.08.022 26297303

[16] Guo Y Xiao Y Zhu H Guo H Zhou Y Shentu Y. Inhibition of Proliferation-Linked Signaling Cascades With Atractylenolide I Reduces Myofibroblastic Phenotype and Renal Fibrosis. Biochem Pharmacol (2021) 183:114344. doi: 10.1016/j.bcp.2020.114344 33221275

[17] Long F Lin H Zhang X Zhang J Xiao H Wang T. Atractylenolide-I Suppresses Tumorigenesis of Breast Cancer by Inhibiting Toll-Like Receptor 4-Mediated Nuclear Factor-kappaB Signaling Pathway. Front Pharmacol (2020) 11:598939. doi: 10.3389/fphar.2020.598939 33363472 PMC7753112

[18] Li Y Wang Y Liu Z Guo X Miao Z Ma S. Atractylenolide I Induces Apoptosis and Suppresses Glycolysis by Blocking the JAK2/STAT3 Signaling Pathway in Colorectal Cancer Cells. Front Pharmacol (2020) 11:273. doi: 10.3389/fphar.2020.00273 32273843 PMC7114890

[19] Wang K Huang W Sang X Wu X Shan Q Tang D. Atractylenolide I Inhibits Colorectal Cancer Cell Proliferation by Affecting Metabolism and Stemness via AKT/mTOR Signaling. Phytomedicine (2020) 68:153191. doi: 10.1016/j.phymed.2020.153191 32135457

[20] Fu XQ Chou JY Li T Zhu PL Li JK Yin CL. The JAK2/STAT3 Pathway is Involved in the Anti-Melanoma Effects of Atractylenolide I. Exp Dermatol (2018) 27(2):201–4. doi: 10.1111/exd.13454 29078004

[21] Long F Wang T Jia P Wang H Qing Y Xiong T. Anti-Tumor Effects of Atractylenolide-I on Human Ovarian Cancer Cells. Med Sci Monit (2017) 23:571–9. doi: 10.12659/msm.902886 PMC5297331 28141785

[22] Huang JM Zhang GN Shi Y Zha X Zhu Y Wang MM. Atractylenolide-I Sensitizes Human Ovarian Cancer Cells to Paclitaxel by Blocking Activation of TLR4/MyD88-Dependent Pathway. Sci Rep (2014) 4:3840. doi: 10.1038/srep03840 24452475 PMC3899591

[23] Ma L Mao R Shen K Zheng Y Li Y Liu J. Atractylenolide I-Mediated Notch Pathway Inhibition Attenuates Gastric Cancer Stem Cell Traits. Biochem Biophys Res Commun (2014) 450(1):353–9. doi: 10.1016/j.bbrc.2014.05.110 24944018

[24] Wynn TA Ramalingam TR. Mechanisms of Fibrosis: Therapeutic Translation for Fibrotic Disease. Nat Med (2012) 18(7):1028–40. doi: 10.1038/nm.2807 PMC3405917 22772564

[25] Vanhove T Goldschmeding R Kuypers D. Kidney Fibrosis: Origins and Interventions. Transplantation (2017) 101(4):713–26. doi: 10.1097/TP.0000000000001608 PMC7228593 27941433

[26] Fujii M Toyoda T Nakanishi H Yatabe Y Sato A Matsudaira Y. TGF-Beta Synergizes With Defects in the Hippo Pathway to Stimulate Human Malignant Mesothelioma Growth. J Exp Med (2012) 209(3):479–94. doi: 10.1084/jem.20111653 PMC3302232 22329991

[27] Mao Z Ma X Rong Y Cui L Wang X Wu W. Connective Tissue Growth Factor Enhances the Migration of Gastric Cancer Through Downregulation of E-Cadherin via the NF-kappaB Pathway. Cancer Sci (2011) 102(1):104–10. doi: 10.1111/j.1349-7006.2010.01746.x 20946117

[28] Finger EC Cheng CF Williams TR Rankin EB Bedogni B Tachiki L. CTGF is a Therapeutic Target for Metastatic Melanoma. Oncogene (2014) 33(9):1093–100. doi: 10.1038/onc.2013.47 PMC3965577 23435419

[29] Jiang CG Lv L Liu FR Wang ZN Na D Li F. Connective Tissue Growth Factor is a Positive Regulator of Epithelial-Mesenchymal Transition and Promotes the Adhesion With Gastric Cancer Cells in Human Peritoneal Mesothelial Cells. Cytokine (2013) 61(1):173–80. doi: 10.1016/j.cyto.2012.09.013 23073116

[30] Liao X Bu Y Jiang S Chang F Jia F Xiao X. CCN2-MAPK-Id-1 Loop Feedback Amplification is Involved in Maintaining Stemness in Oxaliplatin-Resistant Hepatocellular Carcinoma. Hepatol Int (2019) 13(4):440–53. doi: 10.1007/s12072-019-09960-5 PMC6661033 31250351

[31] Wang MY Chen PS Prakash E Hsu HC Huang HY Lin MT. Connective Tissue Growth Factor Confers Drug Resistance in Breast Cancer Through Concomitant Up-Regulation of Bcl-xL and Ciap1. Cancer Res (2009) 69(8):3482–91. doi: 10.1158/0008-5472.CAN-08-2524 19351859

[32] Shimbo A Kajiyama H Tamauchi S Yoshikawa N Ikeda Y Nishino K. Expression of Connective Tissue Growth Factor as a Prognostic Indicator and Its Possible Involvement in the Aggressive Properties of Epithelial Ovarian Carcinoma. Oncol Rep (2019) 42(6):2323–32. doi: 10.3892/or.2019.7352 PMC6826307 31578579

[33] Liu D Fu X Wang Y Wang X Wang H Wen J. Protein Diaphanous Homolog 1 (Diaph1) Promotes Myofibroblastic Activation of Hepatic Stellate Cells by Regulating Rab5a Activity and TGFbeta Receptor Endocytosis. FASEB J (2020) 34(6):7345–59. doi: 10.1096/fj.201903033R PMC7686927 32304339

[34] Leask A. A Centralized Communication Network: Recent Insights Into the Role of the Cancer Associated Fibroblast in the Development of Drug Resistance in Tumors. Semin Cell Dev Biol (2020) 101:111–4. doi: 10.1016/j.semcdb.2019.10.016 31708414

[35] Hutchenreuther J Vincent K Norley C Racanelli M Gruber SB Johnson TM. Activation of Cancer-Associated Fibroblasts is Required for Tumor Neovascularization in a Murine Model of Melanoma. Matrix Biol (2018) 74:52–61. doi: 10.1016/j.matbio.2018.06.003 29885461

[36] Hutchenreuther J Vincent KM Carter DE Postovit LM Leask A. CCN2 Expression by Tumor Stroma Is Required for Melanoma Metastasis. J Invest Dermatol (2015) 135(11):2805–13. doi: 10.1038/jid.2015.279 26168233

[37] Capparelli C Whitaker-Menezes D Guido C Balliet R Pestell TG Howell A. CTGF Drives Autophagy, Glycolysis and Senescence in Cancer-Associated Fibroblasts via HIF1 Activation, Metabolically Promoting Tumor Growth. Cell Cycle (2012) 11(12):2272–84. doi: 10.4161/cc.20717 PMC3383589 22684333

[38] R&D Systems I. Recombinant Human CTGF/CCN2 Protein, Cf (2021). Available at: https://www.rndsystems.com/cn/products/recombinant-human-ctgf-ccn2-protein-cf_9190-cc#product-details.

[39] Wakayama M Abei M Kawashima R Seo E Fukuda K Ugai H. E1A, E1B Double-Restricted Adenovirus With RGD-Fiber Modification Exhibits Enhanced Oncolysis for CAR-Deficient Biliary Cancers. Clin Cancer Res (2007) 13(10):3043–50. doi: 10.1158/1078-0432.Ccr-06-2103 17505007

[40] De Vincenzo A Belli S Franco P Telesca M Iaccarino I Botti G. Paracrine Recruitment and Activation of Fibroblasts by C-Myc Expressing Breast Epithelial Cells Through the IGFs/IGF-1R Axis. Int J Cancer (2019) 145(10):2827–39. doi: 10.1002/ijc.32613 31381136

[41] Invitrogen. TRIzol Reagent User Guide (Pub.No. Man0001271) (2020). Available at: https://assets.thermofisher.cn/TFS-Assets/LSG/manuals/trizol_reagent.pdf.

[42] Schmittgen TD Livak KJ. Analyzing Real-Time PCR Data by the Comparative C(T) Method. Nat Protoc (2008) 3(6):1101–8. doi: 10.1038/nprot.2008.73 18546601

[43] Liu Y Geng YH Yang H Yang H Zhou YT Zhang HQ. Extracellular ATP Drives Breast Cancer Cell Migration and Metastasis via S100A4 Production by Cancer Cells and Fibroblasts. Cancer Lett (2018) 430:1–10. doi: 10.1016/j.canlet.2018.04.043 29733962

[44] Wang Z Wang P Cao L Li F Duan S Yuan G. Long Intergenic Non-Coding RNA 01121 Promotes Breast Cancer Cell Proliferation, Migration, and Invasion via the miR-150-5p/HMGA2 Axis. Cancer Manag and Res (2019) 11:10859–70. doi: 10.2147/cmar.S230367 PMC6941603 31920395

[45] Calvo F Ege N Grande-Garcia A Hooper S Jenkins RP Chaudhry SI. Mechanotransduction and YAP-Dependent Matrix Remodelling Is Required for the Generation and Maintenance of Cancer-Associated Fibroblasts. Nat Cell Biol (2013) 15(6):637–46. doi: 10.1038/ncb2756 PMC3836234 23708000

[46] Yang H Geng YH Wang P Yang H Zhou YT Zhang HQ. Extracellular ATP Promotes Breast Cancer Invasion and Chemoresistance via SOX9 Signaling. Oncogene (2020) 39(35):5795–810. doi: 10.1038/s41388-020-01402-z 32724162

[47] Syed N Chavan S Sahasrabuddhe NA Renuse S Sathe G Nanjappa V. Silencing of High-Mobility Group Box 2 (HMGB2) Modulates Cisplatin and 5-Fluorouracil Sensitivity in Head and Neck Squamous Cell Carcinoma. Proteomics (2015) 15(2-3):383–93. doi: 10.1002/pmic.201400338 PMC4528963 25327479

[48] Yaghobi Z Movassaghpour A Talebi M Abdoli Shadbad M Hajiasgharzadeh K Pourvahdani S. The Role of CD44 in Cancer Chemoresistance: A Concise Review. Eur J Pharmacol (2021) 903:174147. doi: 10.1016/j.ejphar.2021.174147 33961871

[49] Shen YW Zhou YD Chen HZ Luan X Zhang WD. Targeting CTGF in Cancer: An Emerging Therapeutic Opportunity. Trends Cancer (2021) 7(6):511–24. doi: 10.1016/j.trecan.2020.12.001 33358571

[50] Tan TW Lai CH Huang CY Yang WH Chen HT Hsu HC. CTGF Enhances Migration and MMP-13 Up-Regulation via Alphavbeta3 Integrin, FAK, ERK, and NF-kappaB-Dependent Pathway in Human Chondrosarcoma Cells. J Cell Biochem (2009) 107(2):345–56. doi: 10.1002/jcb.22132 19301259

[51] Aguiar DP de Farias GC de Sousa EB de Mattos Coelho-Aguiar J Lobo JC Casado PL. New Strategy to Control Cell Migration and Metastasis Regulated by CCN2/CTGF. Cancer Cell Int (2014) 14:61. doi: 10.1186/1475-2867-14-61 25120383 PMC4130434

[52] Chen PS Wang MY Wu SN Su JL Hong CC Chuang SE. CTGF Enhances the Motility of Breast Cancer Cells via an Integrin-Alphavbeta3-ERK1/2-Dependent S100A4-Upregulated Pathway. J Cell Sci (2007) 120(Pt 12):2053–65. doi: 10.1242/jcs.03460 17550972

[53] Tsai HC Huang CY Su HL Tang CH. CTGF Increases Drug Resistance to Paclitaxel by Upregulating Survivin Expression in Human Osteosarcoma Cells. Biochim Biophys Acta (2014) 1843(5):846–54. doi: 10.1016/j.bbamcr.2014.01.007 24462773

[54] Zeng H Yang Z Xu N Liu B Fu Z Lian C. Connective Tissue Growth Factor Promotes Temozolomide Resistance in Glioblastoma Through TGF-Beta1-Dependent Activation of Smad/ERK Signaling. Cell Death Dis (2017) 8(6):e2885. doi: 10.1038/cddis.2017.248 28617438 PMC5520906

[55] Neesse A Frese KK Bapiro TE Nakagawa T Sternlicht MD Seeley TW. CTGF Antagonism With mAb FG-3019 Enhances Chemotherapy Response Without Increasing Drug Delivery in Murine Ductal Pancreas Cancer. Proc Natl Acad Sci U S A (2013) 110(30):12325–30. doi: 10.1073/pnas.1300415110 PMC3725120 23836645

[56] Tsang M Quesnel K Vincent K Hutchenreuther J Postovit LM Leask A. Insights Into Fibroblast Plasticity: Cellular Communication Network 2 Is Required for Activation of Cancer-Associated Fibroblasts in a Murine Model of Melanoma. Am J Pathol (2020) 190(1):206–21. doi: 10.1016/j.ajpath.2019.09.006 31610176

[57] Deng Q Jiang G Wu Y Li J Liang W Chen L. GPER/Hippo-YAP Signal is Involved in Bisphenol S Induced Migration of Triple Negative Breast Cancer (TNBC) Cells. J Hazard Mater (2018) 355:1–9. doi: 10.1016/j.jhazmat.2018.05.013 29758456

[58] Chen JQ Guo YS Chen Q Cheng XL Xiang GJ Chen MY. Tgfβ1 and HGF Regulate CTGF Expression in Human Atrial Fibroblasts and Are Involved in Atrial Remodelling in Patients With Rheumatic Heart Disease. J Cell Mol Med (2019) 23(4):3032–9. doi: 10.1111/jcmm.14165 PMC6433664 30697920

[59] Shieh JM Tsai YJ Chi JC Wu WB. Tgfβ Mediates Collagen Production in Human CRSsNP Nasal Mucosa-Derived Fibroblasts Through Smad2/3-Dependent Pathway and CTGF Induction and Secretion. J Cell Physiol (2019) 234(7):10489–99. doi: 10.1002/jcp.27718 30426494

[60] Guan X. Cancer Metastases: Challenges and Opportunities. Acta Pharm Sin B (2015) 5(5):402–18. doi: 10.1016/j.apsb.2015.07.005 PMC4629446 26579471

[61] Rowinsky EK Donehower RC. Paclitaxel (Taxol). N Engl J Med (1995) 332(15):1004–14. doi: 10.1056/nejm199504133321507 7885406

