# Supplementary figures and images for: Atractylenolide-I Sensitizes Triple-Negative Breast Cancer Cells to Paclitaxel by Blocking CTGF Expression and Fibroblast Activation
Source: Front Oncol. 2021 Oct 6;11:738534. doi: 10.3389/fonc.2021.738534 (PMC8526898; doi:10.3389/fonc.2021.738534)

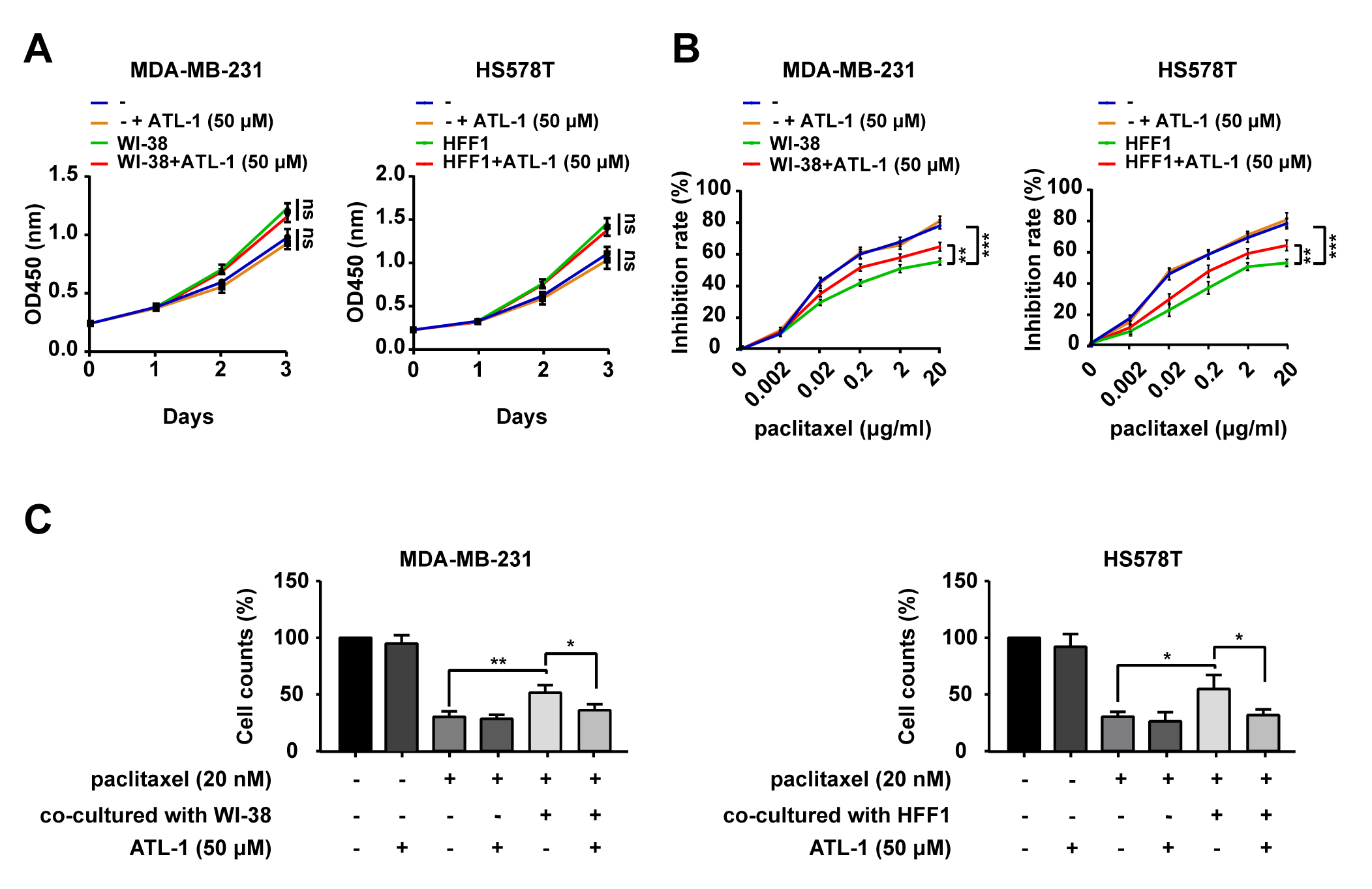

Supplement: Supplementary Figure 1 — ATL-1 increased the sensitivity of tumor cells to paclitaxel. (A) CCK8 assays showed that 50 μM ATL-1 alone had no growth-inhibiting effect on MDA-MB-231 and HS578T cells cultured alone (–) or co-cultured with fibroblasts (HFF1 or WI-38 cells). (B) CCK8 assays showed the growth-inhibiting effects of paclitaxel on MDA-MB-231 and HS578T cells cultured alone (–) or co-cultured with fibroblasts (HFF1 or WI-38 cells) with or without 50 μM ATL-1 treatment for 48 hrs. (C) Trypan blue exclusion assay showed the live cell counts after paclitaxel treatment on MDA-MB-231 and HS578T cells cultured alone or co-cultured with fibroblasts with or without 50 μM ATL-1 treatment for 48 hrs. (A–C) Three technical replicates were performed for each of the three biological replicates. Mean ± SD, *p < 0.05, **p < 0.01, ***p < 0.001 by Student’s t test. [file Image_1.tif]

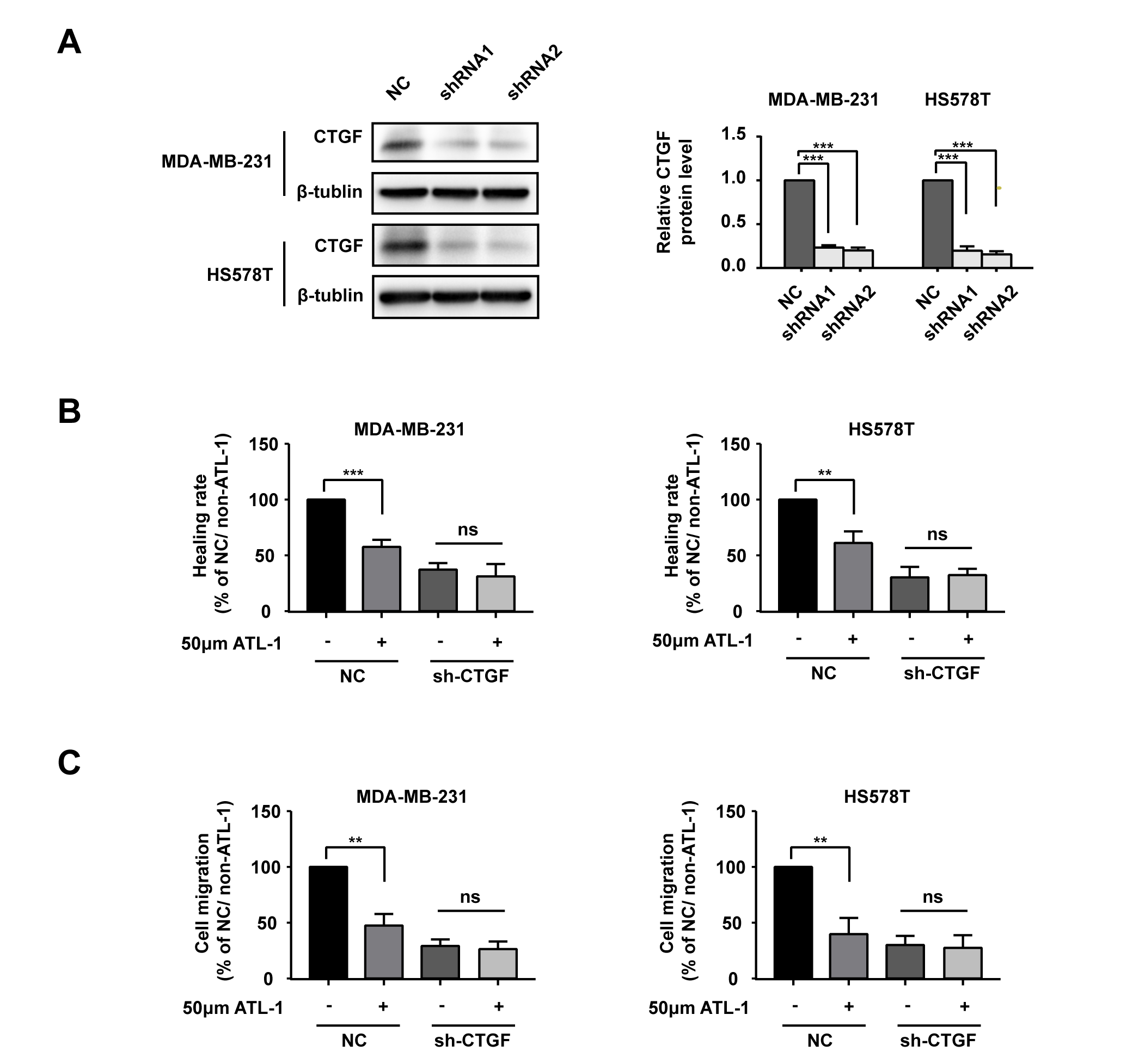

Supplement: Supplementary Figure 2 — ATL-1 inhibits triple-negative breast cancer cell migration via CTGF. (A) Western blotting demonstrated that CTGF expression was knocked down in MDA-MB-231 and HS578T cells. Wound healing (B) and transwell migration (C) assays showed that the reductions in MDA-MB-231 and HS578T cell migration induced by ATL-1 were attenuated by sh-CTGF. Three technical replicates were performed for each of the three biological replicates. Mean ± SD, **p < 0.01, ***p < 0.001 by Student’s t test. [file Image_2.tif]

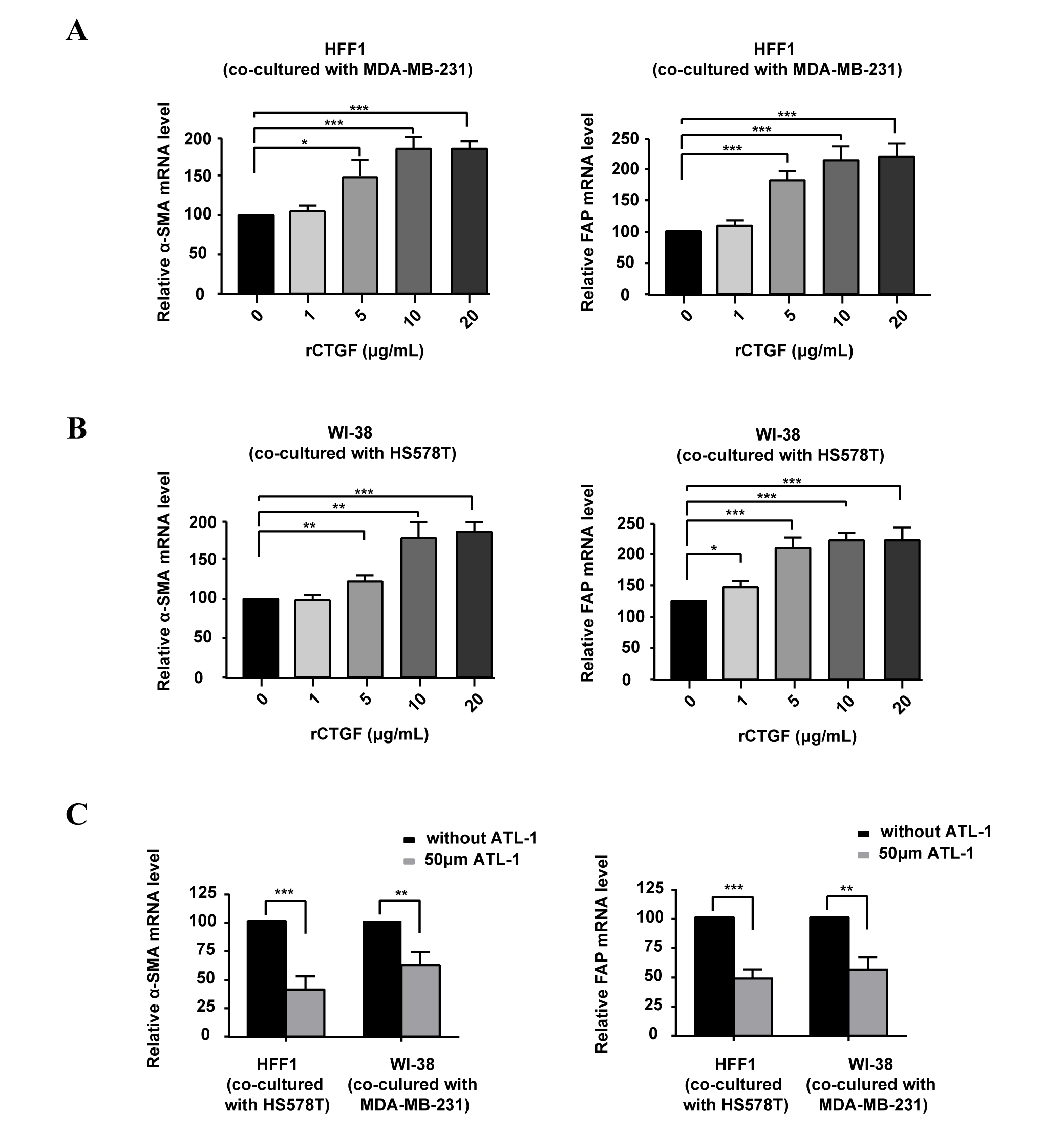

Supplement: Supplementary Figure 3 — The effects of recombinant CTGF (rCTGF) and ATL-1 on the transformation of fibroblasts into CAFs in co-culture systems. (A, B) qRT-PCR demonstrated that treatment with different concentrate of rCTGF in the co-culture system for 48 hrs had different effect on the mRNA levels of CAF markers (FAP and α-SMA) compared with no rCTGF treatment. (C) qRT-PCR demonstrated that treatment with 50 μM ATL-1 in the co-culture system for 48 hrs downregulated the mRNA levels of CAF markers compared with no ATL-1 treatment. Three technical replicates were performed for each of the three biological replicates. Mean ± SD, *p < 0.05, **p < 0.01, ***p < 0.001 by Student’s t test. [file Image_3.tif]

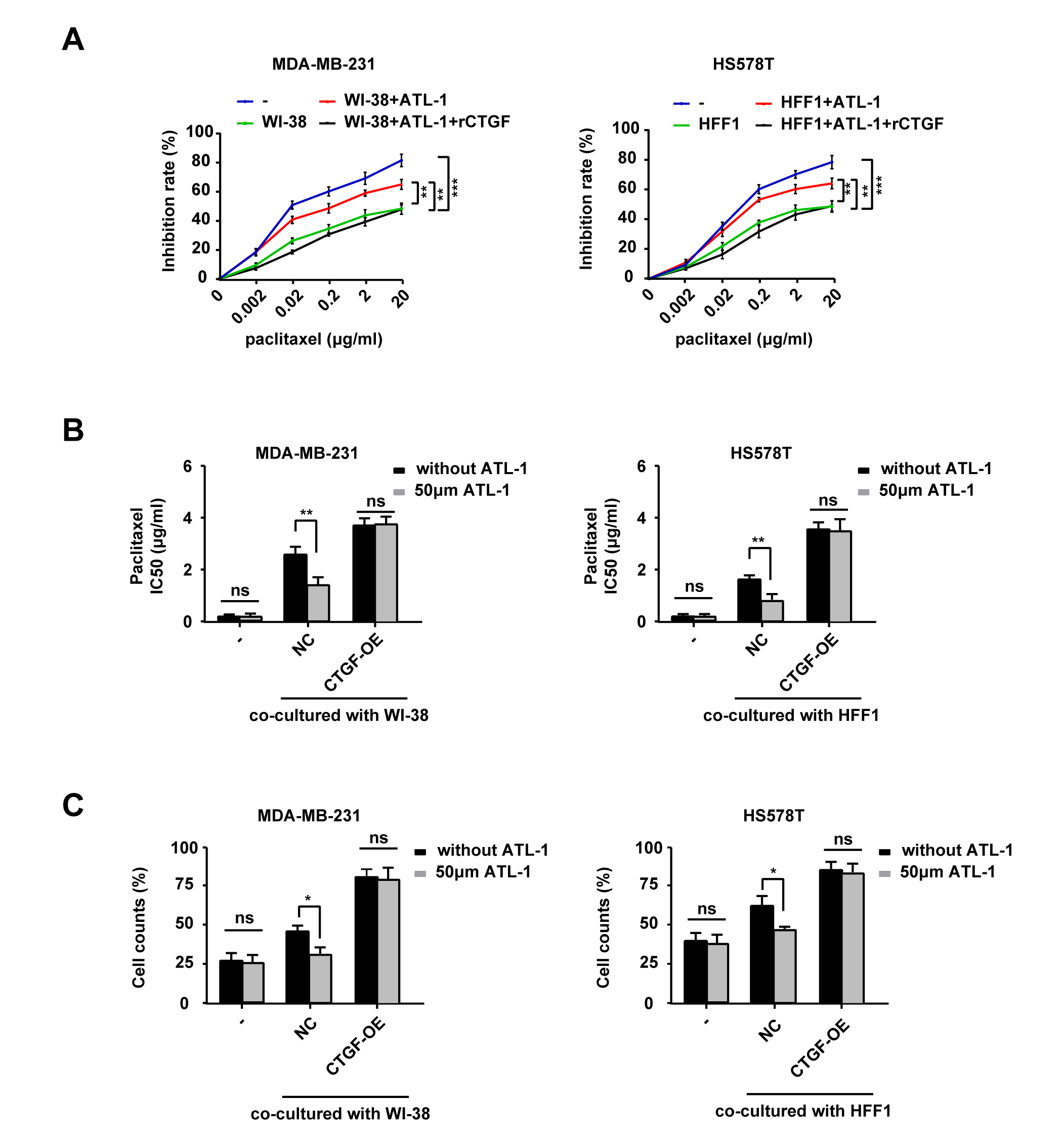

Supplement: Supplementary Figure 4 — ATL-1 increased the sensitivity of TNBC cells to paclitaxel by downregulating the expression of CTGF in fibroblasts. CCK8 (A) assay showed the growth-inhibiting effects of paclitaxel on MDA-MB-231 and HS578T cells cultured alone (–) or co-cultured with fibroblasts with or without ATL-1 treatment and rCTGF treatment for 48 hrs. (B) CCK8 showed the IC50 of paclitaxel on MDA-MB-231 and HS578T cells cultured alone (–) or co-cultured with fibroblasts with or without ATL-1 treatment for 48 hrs. (C) Trypan blue exclusion assay showed the live cell counts after paclitaxel treatment on MDA-MB-231 and HS578T cells cultured alone(–) or co-cultured with fibroblasts with or without 50 μM ATL-1 treatment for 48 hrs. ATL-1 increased the growth-inhibiting effects of paclitaxel in co-culture systems (NC), which was attenuated by the overexpression of CTGF in fibroblasts (CTGF-OE). (A–C) Three technical replicates were performed for each of the three biological replicates. Mean ± SD, *p < 0.05, **p < 0.01, ***p < 0.001 by Student’s t test. [file Image_4.tif]

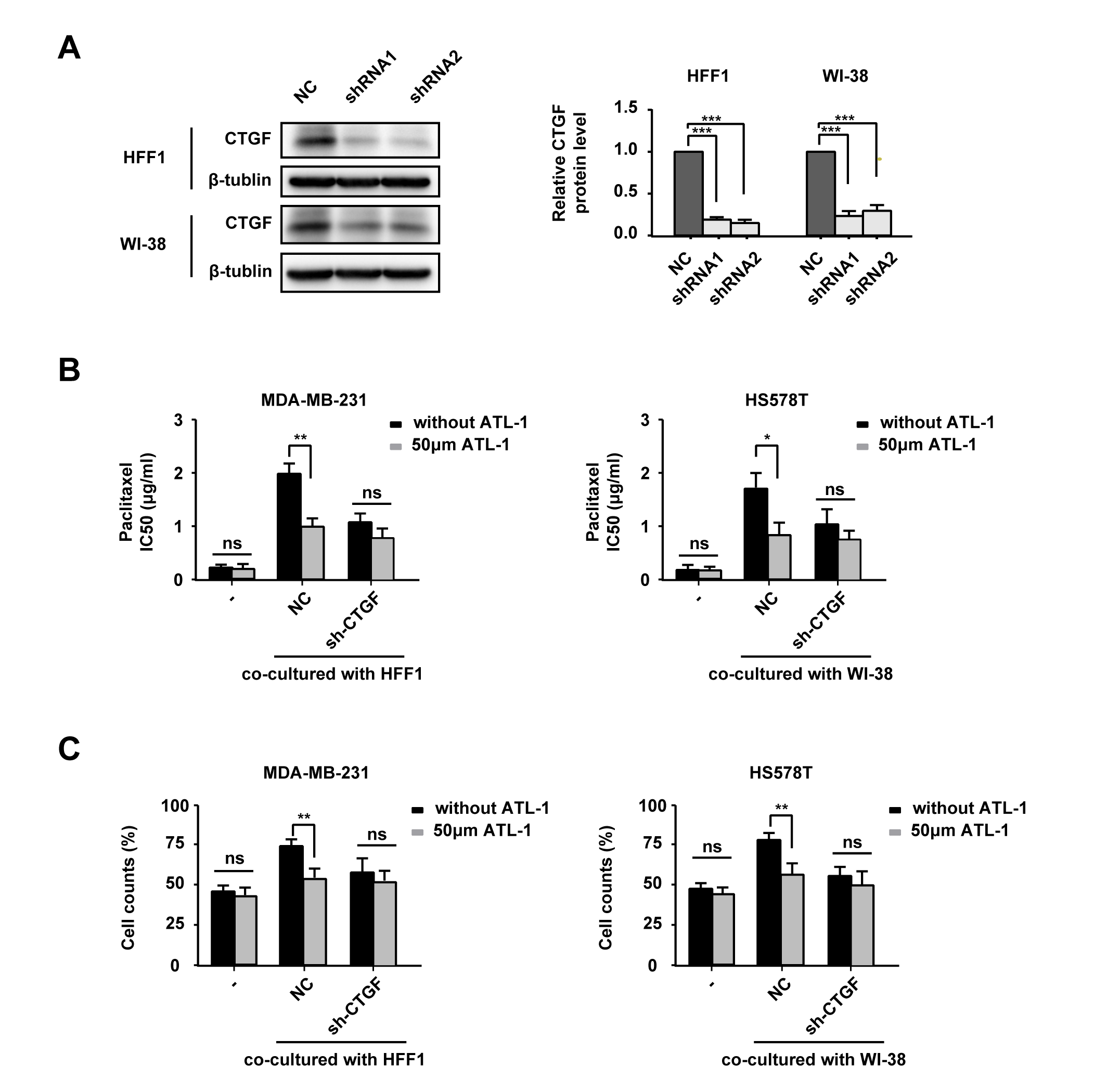

Supplement: Supplementary Figure 5 — ATL-1 increased the sensitivity of TNBC cells to paclitaxel by downregulating the expression of CTGF in fibroblasts. (A) Western blotting demonstrated that CTGF expression was knocked down in HFF1 and WI-38 cells. (B) CCK8 showed the IC50 of paclitaxel on MDA-MB-231 and HS578T cells cultured alone (–) or co-cultured with fibroblasts with or without ATL-1 treatment. (C) Trypan blue exclusion assay showed the cell counts after paclitaxel treatment on MDA-MB-231 and HS578T cells cultured alone (-) or co-cultured with fibroblasts with or without 50 μM ATL-1 treatment. ATL-1 increased the growth-inhibiting effects of paclitaxel in co-culture systems (NC), which was attenuated by sh-CTGF. (A–C) Three technical replicates were performed for each of the three biological replicates. Mean ± SD, *p < 0.05, **p < 0.01, ***p < 0.001 by Student’s t test. [file Image_5.tif]
