# Supplementary material for: Atractylenolide-I Sensitizes Triple-Negative Breast Cancer Cells to Paclitaxel by Blocking CTGF Expression and Fibroblast Activation
Source: Front Oncol. 2021 Oct 6;11:738534. doi: 10.3389/fonc.2021.738534 (PMC8526898; doi:10.3389/fonc.2021.738534)
Supplement: Supplementary file 6 [file Table_1.docx]

**Table S1.** Normalized log2 ratio and p value for each genes in figure 2A

| Gene | Control #1 | Control #2 | Control #3 | ATL-1 #1 | ATL-1 #2 | ATL-1 #3 | *P-value* |
| --- | --- | --- | --- | --- | --- | --- | --- |
| SOX9 | 1.606 | 0.616 | 0.251 | -0.759 | -1.539 | -0.176 | 0.0434 |
| S100P | 0.831 | 1.289 | 0.661 | -1.017 | -0.322 | -1.440 | 0.0079 |
| CD44 | 1.075 | 0.666 | 1.157 | -1.266 | -1.070 | -0.562 | 0.0017 |
| HMGB2 | 1.081 | 0.532 | 1.274 | -1.172 | -0.653 | -1.061 | 0.0021 |
| LOXL2 | 0.739 | 1.268 | 0.940 | -1.167 | -0.808 | -0.973 | 0.0005 |
| CGA | 0.675 | 1.133 | 1.049 | -0.563 | -1.472 | -0.822 | 0.0033 |
| FN1 | 0.688 | 0.906 | 1.235 | -0.952 | -1.444 | -0.433 | 0.0048 |
| KRT16 | 1.069 | 0.710 | 1.181 | -1.127 | -0.855 | -0.978 | 0.0003 |
| CEMIP | 1.112 | 0.882 | 0.997 | -1.076 | -0.937 | -0.977 | <0.0001 |
| CTGF | 1.120 | 1.216 | 0.608 | -0.911 | -1.056 | -0.976 | 0.0005 |
