# Supplementary material for: Atractylenolide-I Sensitizes Triple-Negative Breast Cancer Cells to Paclitaxel by Blocking CTGF Expression and Fibroblast Activation
Source: Front Oncol. 2021 Oct 6;11:738534. doi: 10.3389/fonc.2021.738534 (PMC8526898; doi:10.3389/fonc.2021.738534)
Supplement: Supplementary file 7 [file Table_2.docx]

**Table S2.** Primer list for realtime-PCR

| Gene (*Species*) | Forward primer | Reverse primer |
| --- | --- | --- |
| SOX9 (*Homo sapiens*) | 5’-AGGAAGCTCGCGGACCAGTAC-3’ | 5’-GGTGGTCCTTCTTGTGCTGCAC-3’ |
| S100P (*Homo sapiens*) | 5’-CTCAAGGTGCTGATGGAGAAGG-3’ | 5’-GAACTCACTGAAGTCCACCTGG-3’ |
| CD44 (*Homo sapiens*) | 5’-CCAGAAGGAACAGTGGTTTGGC-3’ | 5’-ACTGTCCTCTGGGCTTGGTGTT-3’ |
| HMGB2 (*Homo sapiens*) | 5’-GGTGAAATGTGGTCTGAGCAGTC-3’ | 5’-CCTGCTTCACTTTTGCCCTTGG-3’ |
| LOXL2 (*Homo sapiens*) | 5’-TGACTGCAAGCACACGGAGGAT-3’ | 5’-TCCGAATGTCCTCCACCTGGAT-3’ |
| CGA (*Homo sapiens*) | 5’-TCCATTCCGCTCCTGATGTGCA-3’ | 5’-CGTCTTCTTGGACCTTAGTGGAG-3’ |
| FN1 (*Homo sapiens*) | 5’-ACAACACCGAGGTGACTGAGAC-3’ | 5’-GGACACAACGATGCTTCCTGAG-3’ |
| KRT16 (*Homo sapiens*) | 5’-CTACCTGAGGAAGAACCACGAG-3’ | 5’-CTCGTACTGGTCACGCATCTCA-3’ |
| CEMIP (*Homo sapiens*) | 5’-ACCGAGCACATTCCAACTACCG-3’ | 5’-GGCAGAGATGATTGAGAGGAACG-3’ |
| CTGF (*Homo sapiens*) | 5’-CTTGCGAAGCTGACCTGGAAGA-3’ | 5’-CCGTCGGTACATACTCCACAGA-3’ |
| GAPDH (*Homo sapiens*) | 5’-GTCTCCTCTGACTTCAACAGCG-3’ | 5’-ACCACCCTGTTGCTGTAGCCAA-3’ |
