# Supplementary material for: Atractylenolide-I Sensitizes Triple-Negative Breast Cancer Cells to Paclitaxel by Blocking CTGF Expression and Fibroblast Activation
Source: Front Oncol. 2021 Oct 6;11:738534. doi: 10.3389/fonc.2021.738534 (PMC8526898; doi:10.3389/fonc.2021.738534)
Supplement: Supplementary file 8 [file Table_3.docx]

**Table S3.** The sequences of shRNAs

| Gene-specific shRNAs | Sequence |
| --- | --- |
| CTGF shRNA1 | 5’- TCTTTGAATCGCTGTACTActcgagTAGTACAGCGATTCAAAGA -3’ |
| CTGF shRNA2 | 5’- GAACATTAAGAAGGGCAAActcgagTTTGCCCTTCTTAATGTTC -3’ |
